# Alveolar macrophages in early stage COPD show functional deviations with properties of impaired immune activation

**DOI:** 10.3389/fimmu.2022.917232

**Published:** 2022-07-28

**Authors:** Kevin Baßler, Wataru Fujii, Theodore S. Kapellos, Erika Dudkin, Nico Reusch, Ari Horne, Benedikt Reiz, Malte D. Luecken, Collins Osei-Sarpong, Stefanie Warnat-Herresthal, Lorenzo Bonaguro, Jonas Schulte-Schrepping, Allon Wagner, Patrick Günther, Carmen Pizarro, Tina Schreiber, Rainer Knoll, Lisa Holsten, Charlotte Kröger, Elena De Domenico, Matthias Becker, Kristian Händler, Christian T. Wohnhaas, Florian Baumgartner, Meike Köhler, Heidi Theis, Michael Kraut, Marc H. Wadsworth, Travis K. Hughes, Humberto J. Ferreira, Emily Hinkley, Ines H. Kaltheuner, Matthias Geyer, Christoph Thiele, Alex K. Shalek, Andreas Feißt, Daniel Thomas, Henning Dickten, Marc Beyer, Patrick Baum, Nir Yosef, Anna C. Aschenbrenner, Thomas Ulas, Jan Hasenauer, Fabian J. Theis, Dirk Skowasch, Joachim L. Schultze

**Affiliations:** ^1^ Genomics and Immunoregulation, Life & Medical Sciences (LIMES) Institute, University of Bonn, Bonn, Germany; ^2^ Computational Life Sciences, Life & Medical Sciences (LIMES) Institute, University of Bonn, Neuherberg, Germany; ^3^ Comma Soft AG, Bonn, Germany; ^4^ Helmholtz Zentrum München - German Research Center for Environmental Health, Institute of Computational Biology, Neuherberg, Germany; ^5^ Immunogenomics & Neurodegeneration, German Center for Neurodegenerative Diseases and the University of Bonn, Bonn, Germany; ^6^ PRECISE Platform for Single Cell Genomics and Epigenomics, German Center for Neurodegenerative Diseases (DZNE) and the University of Bonn, Bonn, Germany; ^7^ Systems Medicine, German Center for Neurodegenerative Diseases (DZNE), Bonn, Germany; ^8^ Department of electrical engineering and computer science, University of California, Berkeley, CA, United States; ^9^ Center for computational biology, University of California, Berkeley, CA, United States; ^10^ Department of Internal Medicine II, University Hospital Bonn, Section of Pneumology, Bonn, Germany; ^11^ Prevention, Aging & Systems Immunology, Deutsches Zentrum für Neurodegenerative Erkrankungen (DZNE), Bonn, Germany; ^12^ Computational Biology, Boehringer Ingelheim Pharma GmbH & Co. KG, Biberach, Germany; ^13^ Institute for Medical Engineering & Science, Department of Chemistry, and Koch Institute for Integrative Cancer Research, Massachusetts Institute of Technology, Cambridge, MA, United States; ^14^ Broad Institute of MIT and Harvard; Ragon Institute of MGH, MIT and Harvard, Cambridge, MA, United States; ^15^ Institute of Structural Biology, University Hospital, University of Bonn, Bonn, Germany; ^16^ Biochemistry & Cell Biology of Lipids, Life & Medical Sciences (LIMES) Institute, University of Bonn, Bonn, Germany; ^17^ University Clinics for Radiology, University Hospital Bonn, Bonn, Germany; ^18^ Translational Medicine & Clinical Pharmacology, Boehringer Ingelheim Pharma GmbH & Co. KG, Biberach, Germany; ^19^ Chan-Zuckerberg Biohub, San Francisco, CA, United States; ^20^ Ragon Institute of MGH, MIT, and Harvard, Cambridge, MA, United States; ^21^ Department of Internal Medicine and Radboud Center for Infectious Diseases (RCI), Radboud University Medical Center, Nijmegen, Netherlands; ^22^ School of Life Sciences Weihenstephan, Technical University of Munich, Munich, Germany, Department of Mathematics, Technical University of Munich, Munich, Germany

**Keywords:** chronic obstructive pulmonary disease, bronchoalveolar lavage, blood, macrophage, monocyte, impaired immune activation, TGF-β1

## Abstract

Despite its high prevalence, the cellular and molecular mechanisms of chronic obstructive pulmonary disease (COPD) are far from being understood. Here, we determine disease-related changes in cellular and molecular compositions within the alveolar space and peripheral blood of a cohort of COPD patients and controls. Myeloid cells were the largest cellular compartment in the alveolar space with invading monocytes and proliferating macrophages elevated in COPD. Modeling cell-to-cell communication, signaling pathway usage, and transcription factor binding predicts TGF-β1 to be a major upstream regulator of transcriptional changes in alveolar macrophages of COPD patients. Functionally, macrophages in COPD showed reduced antigen presentation capacity, accumulation of cholesteryl ester, reduced cellular chemotaxis, and mitochondrial dysfunction, reminiscent of impaired immune activation.

## Introduction

Worldwide, chronic obstructive pulmonary disease (COPD) is the third leading cause of death (1, 2). Due to smoking and increasing air pollution, the current prevalence of 10.1% is estimated to further increase in the next decades (2). Considering the enormous medical and financial burden of COPD, there is a need to develop efficient biomarker-based diagnostics, and molecularly guided therapies. It is now accepted that COPD is a heterogeneous disease manifesting as a clinical syndrome with structural pulmonary abnormalities, lung function impairment, chronic respiratory symptoms, or any combination of these. Consequently, the pathogenesis of the disease is complex with numerous co-existing mechanisms with inflammation being one of the most prominent and important mechanisms (3). Lung inflammation in COPD is characterized by alterations in the number and function of immune cells. Alveolar macrophages (AMs) are considered to be one of the major orchestrators (4). Yet, little is known about the heterogeneity of AMs in COPD as well as the underlying molecular mechanisms leading to AM alterations, particularly during earlier disease stages.

To characterize molecular and functional alterations in the myeloid compartment in COPD, we here applied single-cell transcriptomics combined with extended data analytics, as well as phenotypic and functional assays to characterize the molecular changes in myeloid cells derived from bronchoalveolar lavage fluid (BALF) and peripheral blood obtained from patients with early-stage COPD (Global Initiative for Chronic Obstructive Lung Disease (GOLD) stage 2).

## Results

### Heterogeneous cellular states of macrophages in the human alveolar space

**Figure 1:**
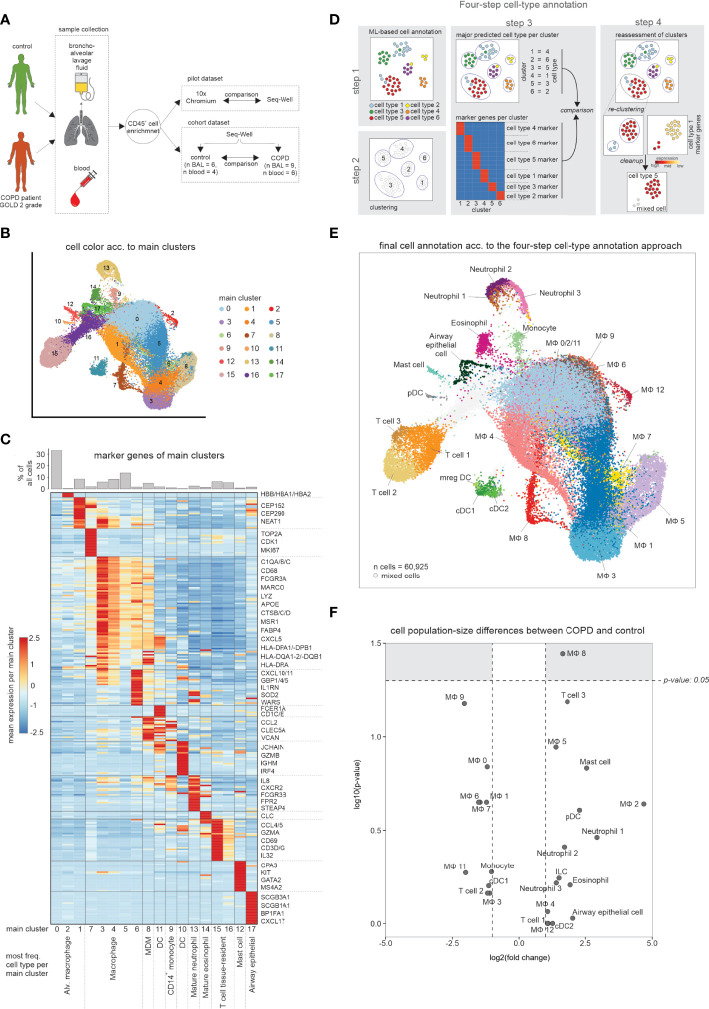
scRNA-seq data of BALF samples obtained from COPD patients and healthy controls. **(A)** Schematic workflow of the present study. Bronchoalveolar lavage fluid (BALF) and peripheral blood was obtained from control donors and COPD patients (GOLD stage 2). After enrichment for immune cells (CD45+ cells), single-cell RNA-seq was performed. **(B)** UMAP representation of integrated BALF data obtained from all COPD patients and control donors. Coloring and numbering according to identified main clusters. **(C)** Heatmap of the calculated marker genes per main cluster with a bar chart representation of the relative cell type proportions at the top. The marker gene expression per cluster is represented as a z-transformed value (across all clusters). Rows of the heatmap are clustered hierarchically. At the bottom of the plot, the main cell type is displayed, which is contained in the respective main cluster. **(D)** Schematic workflow of the four-step annotation approach, including machine learning-based cell type annotation, clustering, assignment and subsequent confirmation of a cluster to a cell type according to the machine learning-based cell type annotation, and identification of ‘contaminating’ cells (referred to as ‘mixed cells’). **(E)** Final cell type annotation of integrated BALF data according to the four-step annotation approach. **(F)** Volcano plot visualization of log2 fold changes and negative log10 p-values (Wilcoxon rank sum test) of changes in cell type occurrence in BALF of samples obtained from COPD patients and controls. BALF, bronchoalveolar lavage fluid; alv., alveolar; MDM, monocyte-derived macrophage; DC, dendritic cell; n, number; MФ, macrophage.

We obtained freshly isolated BALF material and peripheral blood (**Figure 1A**) from COPD patients and donors with chronic cough, but without any signs for pathophysiological alterations of the lung (hereafter referred to as ‘control’) (**Table S1**). We conducted a pilot experiment, in which we obtained single-cell RNA-sequencing (scRNA-seq) data using the most widely used droplet-based solution [Chromium from 10x Genomics (5)] and a well-based method [Seq-Well (6)]. After identification of cell-types based on marker gene expression of defined clusters (**Figure S1A**), we compared the cell populations between the two technologies. As ground truth, we characterized the cellular compartment in the alveolar space using multi-color flow cytometry (MCFC) (**Table S2**, see **methods**). All three approaches identified macrophages as the predominant cell type in the alveolar space (**Figure S1B**). When determining the cell type distribution for the droplet- and well-based scRNA-seq methods, granulocytes (neutrophils, eosinophils) were almost undetectable in the droplet-based method (**Figure S1B**).

Since the population structure in Seq-Well was more closely related to MCFC data, we continued with the well-based scRNA-seq method and generated 60,925 single-cell transcriptomes from BALF derived from 9 patients with early-stage COPD (GOLD stage 2) and 6 controls, as well as 54,569 single-cell transcriptomes from peripheral blood of 6 COPD patients and 4 controls (**Figure 1A**; **Table S1**). Starting with BALF cells, we first used a classical clustering approach and visualized the data *via* UMAP in 17 clusters (**Figure 1B**). By marker gene identification on the majority of cells in each of the clusters, we identified the major cell types present in BALF (**Figure 1C**). A more detailed inspection of individual clusters revealed further cluster substructures. To better describe the cellular compartment in BALF we developed and applied a four-step cell type annotation procedure (**Figure 1D**, **S1C–G**) (for details see methods section *‘four-step cell type annotation’*). Macrophages were not only the most prevalent, but also the most heterogeneous class of immune cells in the alveolar space (**Figure 1E**, **Table S3**), but we also identified dendritic cells (DCs), monocytes, neutrophils, eosinophils, mast cells and T cells in BALF which is in line with recent reports (7). Determination of relative frequencies between COPD and control revealed one of the macrophage states (MФ8) to be elevated in COPD, while the majority of cell types and states did not significantly differ between the COPD and control group (**Figure 1F**). Collectively, single-cell transcriptomics reveals a heterogeneous landscape of myeloid cells in BALF with slight shifts in cell state distributions between early stage COPD (GOLD stage 2) and controls.

### Proliferating and monocyte-like macrophage states are elevated in COPD

**Figure 2:**
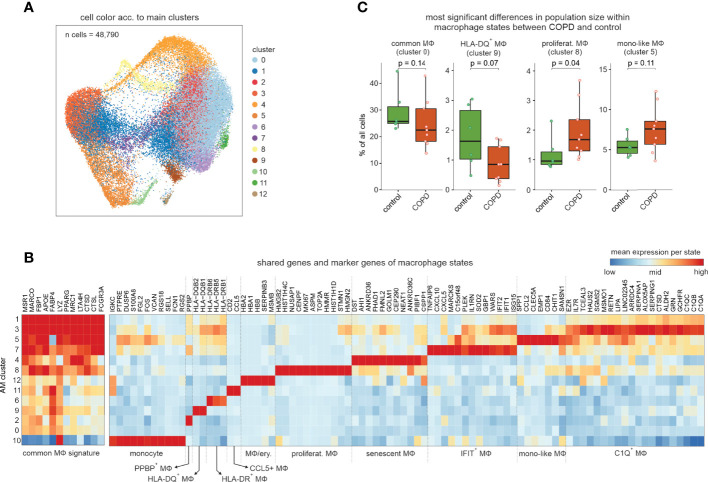
Exploration of the macrophage and monocyte cell types and states in human BALF. **(A)** UMAP representation and clustering of cells annotated as monocytes or macrophages by the four-step annotation approach (according to **Figure 1**). **(B)** Heatmap of marker genes per macrophage/monocyte cluster (referred to as ‘macrophage states’; according to **Figure 2A**). The marker gene expression per macrophage state is represented as a z-transformed value across all macrophage states. On the left side of the heatmap, conserved macrophage markers are depicted. Columns and rows of the heatmap are sorted by hierarchical clustering. **(C)** Box visualization plot (with marked median values) of most significant differences in population sizes within the identified macrophage states between COPD and control (error bars indicating the standard deviation; statistics based on Wilcoxon rank sum test). n, number; MФ, macrophage.

To further characterize the most prevalent and heterogeneous cell types in the alveolar space, we subclustered macrophages and monocytes excluding non-immune cells, neutrophils, basophils, eosinophils, mast cells, DCs, and T cells, which resulted in a total of 13 clusters (**Figures 2A, B** **)**. Except for cluster 10 (monocytes), all other clusters expressed macrophage cell lineage markers (*MSR1*, *MRC1*, *MARCO).* BALF-derived macrophages displayed remarkable transcriptional plasticity. The MФ8 macrophage state, elevated in COPD, was characterized by proliferation-associated genes (*MKI67*, *TOP2A*, and *NUSAP1*), as well as increased expression of histone genes (*HIST1H4C* and *HIST1H1D*) and most of the MФ8 cells were computationally assigned to the G2/M cell cycle phase (**Figure S2A**), strongly supporting these cells representing proliferating macrophages. MФ6 and MФ9 macrophage states were highly enriched for major histocompatibility class (MHC) II expression (*HLA-DQ* and *HLA-DR* respectively), while the MФ12 cell state carried hemoglobin genes (*HBA2*, *HBA1*, and *HBB*) either due to engulfed erythrocytes or induction of hemoglobin genes in macrophages.

Except for the macrophage states MФ12, 2 and 11, we did not identify any donor effect (**Figure S2B**) with the latter being characterized by *CCL5* expression (CCL5+ macrophage state). The MФ5 state exhibited relatively strong expression of the monocyte-associated genes *VCAN* and *S100A8* together with the monocyte attractant *CCL2* and the late monocyte-to-macrophage differentiation marker *CHIT1* and was therefore designated as ‘monocyte-like’ (mono-like) macrophages. Furthermore, these cells, together with the proliferating macrophages, exhibited the largest relative increase in population size in COPD (**Figure 2C**). The monocyte-like macrophages also shared some markers with the MФ7 cell state, which was additionally high in interferon-response genes (*IFIT1* and *IFIT2*), and MФ3 cells characterized by increased expression of complement components (*C1QA-C*) and alpha-1-antitrypsin (*SERPINA1*).

Next, we predicted the functions of each macrophage state by gene set variation analysis (GSVA) (**Figure S2C, D** **)**, illustrating shared, but also cluster-specific functions. Among the shared terms, we found enrichment of ‘antigen presentation’, ‘endocytosis’, ‘oxidative phosphorylation’ and ‘β-oxidation’, which reflect some of the basic cellular processes of macrophages in the alveolar space. Intriguingly, the MФ4 cell state revealed a specific enrichment of the mTOR signaling pathway, which was described to be associated with cellular senescence in non-immune cells from the lung (8). This was further corroborated by enrichment analysis of gene sets associated with cellular senescence, namely genes associated with cell aging and mitochondrial functions (**Figure S2E**). Furthermore, a senescent molecular phenotype of the MФ4 cell state was supported by downregulation of the genes also downregulated in the recently described IMM-age signature derived from aged immune cells (9) (**Figure S2E**). Collectively, macrophages in BALF exist in numerous different molecular and functional states with proliferating and ‘monocyte-like’ macrophage states being elevated in COPD.

### Altered lipid metabolism and stressed macrophage phenotypes in COPD

**Figure 3:**
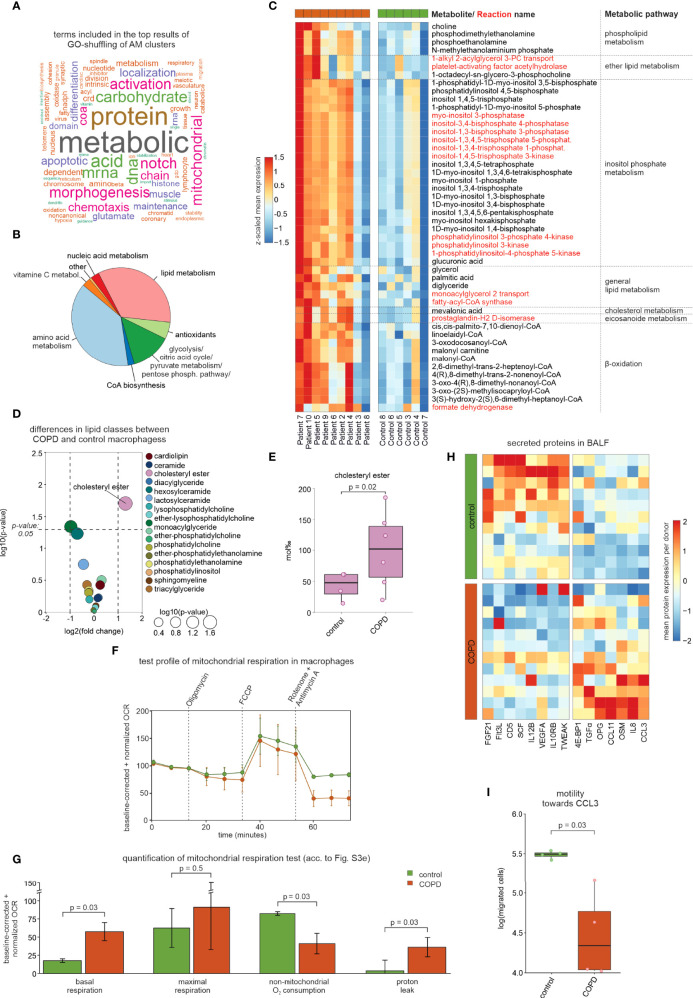
Modeling of the metabolic landscape and alterations in macrophages. **(A)** Word cloud of the most common words in the top predicted terms of the GO-shuffling approach across all macrophage clusters. **(B)** Compass results of the modeled metabolic landscape in macrophages. The pie chart summarizes and categorizes the predicted metabolites and pathways that are significantly different between COPD and control. **(C)** Heatmap showing the metabolites and pathways that were predicted by Compass as altered in COPD and that were associated with lipid metabolism. Metabolites are shown in black and reactions in red. Columns and rows of the heat map are sorted by hierarchical clustering. **(D)** Volcano plot visualization of log2 fold changes and negative log10 p-values (Wilcoxon rank sum test) of lipid class levels between COPD and control macrophages obtained by lipidomics analysis. **(E)** Box plot with marked median values of cholesteryl ester proportions with the representation of individual donors. **(F)** Evaluation of mitochondrial function *via* the time-dependent course of the oxygen consumption rate (OCR) in macrophages using baseline-corrected values. Error bars indicate the standard deviations (control n = 2, COPD n = 3). Dashed arrows represent the injection of various compounds (shown at the top of the plot) used to assess different aspects of mitochondrial function (according to **Figure S3** **E**). **(G)** Bar plots showing quantifications of different aspects of mitochondrial function inferred from the OCR measurement in **Figure 3F** (according to **Figure S3** **E**; error bars indicating the standard deviation; statistics based on t-test). **(H)** Heatmap representation of proteins detected in BALF with a p-value < 0.1 according to the Wilcoxon rank sum test between COPD patients and control donors (control n = 11, COPD n = 12). The mean protein expression (identified by Olink Proteomics) per donor is represented as a z-transformed value (across all donors). Columns of the heatmap are sorted by hierarchical clustering. **(I)** Quantification of the migratory capability of macrophages towards CCL3 displayed in a box plot with marked median values and the representation of individual donors (control n = 4, COPD n = 4; error bars indicating the standard deviation; statistics based on t-test). BALF, bronchoalveolar lavage fluid; OCR, oxygen consumption rate.

To determine overall functional differences between control and COPD based on macrophage state information, we developed ‘GO-shuffling’ as a GO enrichment approach (**Figure S3A**, see methods section *‘Gene set distance analysis of annotated cell types’* for more detail). This enrichment analysis showed that mainly metabolism-associated terms contributed to the separation of COPD patients from control donors (**Figure 3A**). To examine potential COPD-associated changes in metabolism, we applied the Compass algorithm (10) to comprehensively model the metabolic differences between COPD and control macrophage states. The largest differences were found in amino acid and lipid metabolism (**Figure 3B**), with an overall higher predicted metabolic activity in COPD samples (**Figure 3C**). Among the differential lipid-associated metabolites and reactions, phosphorylation of inositol was most prominent, but we also found altered metabolites and reactions, indicating increased transport (monoacylglycerol), synthesis (phospholipids and cholesterol) and degradation (β-oxidation) of lipids in COPD macrophages. In concordance with the increase in lipid metabolism in COPD patients (predicted by Compass), we observed an overall higher expression of genes found in lipid-associated gene sets that were contained in the top 1% of the functional gene sets (**Figure S3B**). Among these genes, we found several receptors for cholesterol uptake (*CD36*, *LDLR*, *MSR1*, and *TREM2*) and genes of cholesterol storage mediated by cholesteryl ester synthesis (*ACAT1/2* and *SOAT1*), but also genes associated with cholesteryl ester hydrolases (*LIPA*, *CES1*, and *NCEH1*) (**Figure S3B, C** **)**. Next, we validated the *in silico* prediction of altered lipid metabolism in COPD by performing lipidomics analyses of 229 lipid species in macrophages obtained either from COPD GOLD 2 patients or control donors. We observed the greatest difference in the lipid class of cholesteryl esters, which was significantly higher in COPD macrophages than in controls (**Figure 3D, E** **)**. These findings indicate that the macrophages in COPD patients show a pulmonary foam cell-like response, which has been reported for other lung diseases, such as pulmonary alveolar proteinosis (11). This cellular phenotype is characterized by the cells being predominantly cholesterol-laden. The accumulation of cholesterol in macrophages of pulmonary alveolar proteinosis patients has been associated with downregulated expression of the cholesterol transporter *ABCG1* (12). Furthermore, the accumulation of cholesteryl ester has been described in microglia as a consequence of deficient TREM2 signaling (13). Surprisingly, we found an upregulation of *ABCG1* and *TREM2* expression in COPD macrophages (**Figure S3B**). However, NOTCH signaling was also predicted as a strong separator of COPD and control macrophages (**Figure 3A**) and, as a consequence, might result in perturbed TREM2 signaling. Investigation of this gene set revealed increased expression levels of the metalloprotease-disintegrins *ADAM10* and *ADAM17* and the γ-secretase component *APH1A* (**Figure S3D**). These enzymes can cleave TREM2 from the surface and thus interfere with the downstream signal transmission (14). It is possible that elevated TREM2 expression in macrophages is a consequence of COPD-mediated tissue damage and thus increased cellular stress.

Since both increased metabolic activity (**Figure 3C**) and putative cell stress demand high amounts of energy, we hypothesized that energy turnover might be increased in macrophages from COPD patients and therefore investigated the mitochondrial function of AMs. In three COPD patients and 2 control donors, we were able to isolate sufficient numbers of viable cells to measure mitochondrial function. Indeed, we observed an increased baseline respiration rate in macrophages derived from COPD patients (**Figure 3F, G**, **Figure S3E**), which reflects an elevated energy demand. In line with previous reports (15), we found a significant increase in proton leakage in COPD macrophages, despite similar levels of ATP production, which is indicative for mitochondrial dysfunction and increased ROS production in COPD (16).

Reduction of chemotaxis was also predicted for COPD macrophages (**Figure 3A**). While CCL3 was elevated in BALF from COPD patients (**Figure 3H**), the chemotaxis of COPD macrophages towards CCL3 was reduced (**Figure 3I**), indicating that single-cell transcriptomes indeed correctly predicted macrophage function, while elevated chemokine levels in BALF did not serve as a surrogate for cellular function. Taken together, the heterogeneous landscape of BALF-derived macrophages is linked to numerous molecular and cellular alterations in COPD, of which we highlight metabolic and chemotactic changes together with evidence of pronounced cellular stress.

### COPD leads to downregulation of MHC expression

**Figure 4:**
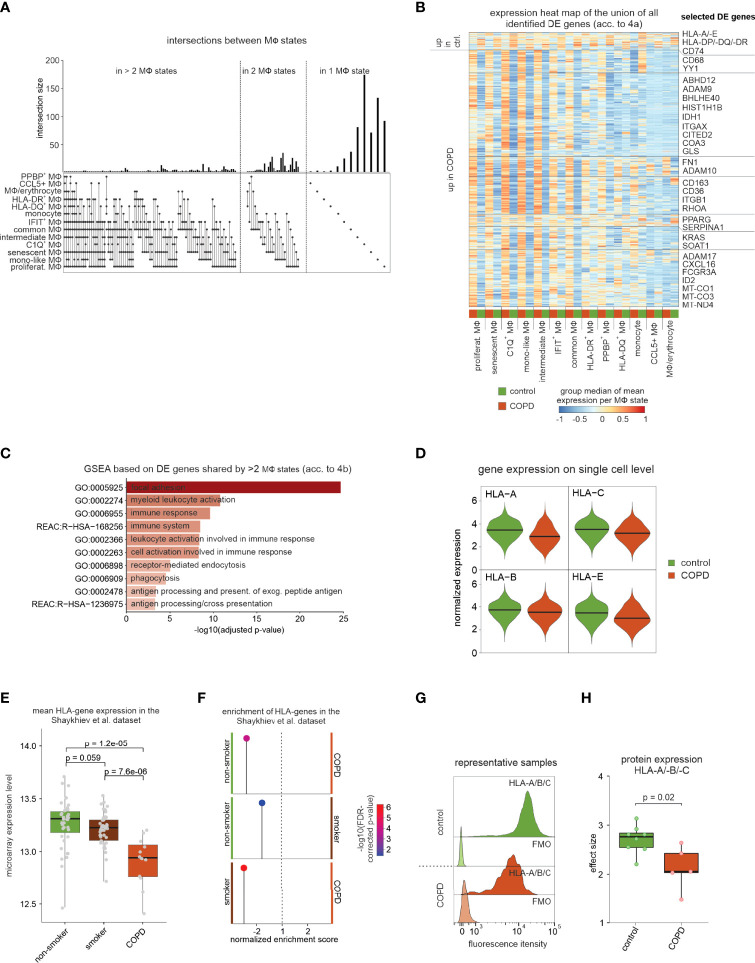
DE gene analysis of identified macrophage states. **(A)** UpSet plot of calculated DE genes across macrophage states. DE genes found in the same states are binned and the size of the bins is represented as a bar chart. At the bottom, dots indicate which macrophage states contained and shared these DE genes. **(B)** Heat map representation of the union of all DE genes found in the macrophage states. Depicted is the group median (group = COPD or control) of the z-transformed mean expression data per donor and macrophage state across all macrophage states, and the names of some selected DE genes are shown on the right side of the plot. Columns and rows of the heat map are sorted by hierarchical clustering. **(C)** Selected functional gene sets from GSEA based on DE genes that reach the defined significance cutoffs for more than two macrophage states (acc. to **Figure 4B**). **(D)** Violin plot with marked median of HLA-A/-B/-C and -E expression in all macrophages based on scRNA-seq data. The plot shows the expression across the donors, whereby the donors were downsampled to the same number of cells, followed by downsampling to the same number of cells between COPD and control. The plot displays cells with an expression > 0. **(E)** Box plots (with marked median values) showing the mean expression per sample of HLA genes expressed in macrophages (error bars indicating the standard deviation; statistics are based on the Wilcoxon rank sum test). The data are obtained from Shaykhiev et al. (17). **(F)** Pin plot representing the enrichments in the samples of Shaykhiev et al. of HLA genes expressed in macrophages. **(G)** Fluorescence intensity histograms showing representative samples of flow cytometric analysis of HLA-A/-B/-C expression on the cell surface of isolated macrophages (FMO = fluorescence minus one). **(H)** Box plots with marked median of the calculated effect sizes of HLA-A/-B/-C expression in COPD and control with the representation of individual donors (control n = 8, COPD n = 5; error bars indicating the standard deviation; statistics based on Wilcoxon rank sum test). MФ, macrophage; mono, monocyte; DE, differentially expressed; GSEA, gene set enrichment analysis; FDR, false discovery rate; FMO, fluorescence minus one.

We next intended to determine differential gene expression across different macrophage states between COPD and controls. Here, we applied an approach, which includes patient information by testing all possible pairs of patients and controls followed by utilizing the median Wilcoxon score of the pairwise tests as a test statistic (**Figure S4A–D**, for more detail see methods section ‘*Distribution-free DE analysis across patient groups’*). Visualization of the DE genes per macrophage state shows that the majority of the observed transcriptional differences are macrophage state-specific (**Figure 4A**), albeit trends for differential expression in the same direction were often seen for other macrophage states as well (**Figure 4B**). Interestingly, transcriptional differences are mainly attributable to increased expression in COPD.

In accordance with the Compass analysis (**Figure 3**), lipid metabolism-associated genes (*e.g. CD36*, *COLEC12*, *SOAT1*, and *PPARG*) were identified to be upregulated in COPD (**Figure 4B**). Further, metalloprotease-disintegrins *ADAM9*, *ADAM10* and *ADAM17*, as well as the surface molecule *CD163* were elevated across many macrophage states in COPD, which corroborates earlier findings for CD163 by immunohistochemistry (18) (**Figure 4B** **)**. Gene set enrichment analysis (GSEA) revealed terms associated with focal adhesion and antigen processing and presentation (**Figure 4C**).

When plotting the expression of the top expressed MHC class I-encoding genes (*HLA-A*, *HLA-B*, *HLA-C*, *HLA-E*) (**Figure 4D**) and MHC II-encoding genes (*HLA-DRA*, *HLA-DRB1*, *HLA-DRB5*, *HLA-DPA1*, *HLA-DPB1*, and *HLA-DQB1*) (**Figure S4E**), we found these genes largely to be downregulated in COPD. We identified similar downregulation of MHC-encoding gene expression in bulk transcriptome data (17) comparing BALF-derived macrophages from healthy donors, healthy smokers, and COPD patients (**Figures 4E** **, F**). Downregulation of MHC molecules was most pronounced in COPD and thus not solely due to smoking. Next, we isolated BALF macrophages from additional patients and measured surface protein levels of MHC class I (HLA-A/-B/-C) (**Figures 4G** **, H**) and class II (HLA-DR) (**Figure S4F** **, G**). MHC class I was significantly reduced on macrophages derived from COPD patients, while MHC class II molecules only showed a trend towards lower expression. In summary, DEG expression analysis revealed significant transcriptional changes in macrophages, including the downregulation of MHC I-encoding gene expressions, which was also apparent on protein level.

### Cell-to-cell communication *via* TGF-β signaling explains changes in macrophage states

**Figure 5:**
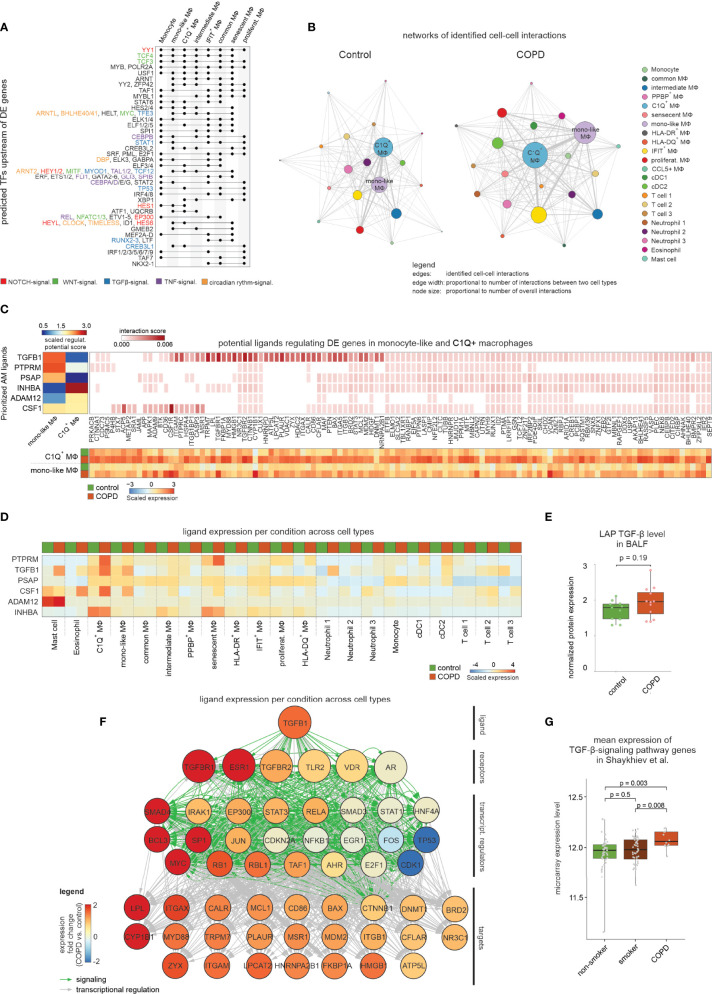
Modeling the cell-to-cell interactions of BALF cells. **(A)** UpSet plot of predicted transcriptional regulators of DE genes. Dots indicate which clusters contain and share predicted transcriptional regulators. The names of selected regulators are shown on the right side of the plot with the font color indicating the association with NOTCH, WNT, TGF-β1, TNF or circadian rhythm signaling. **(B)** Network representation of predicted cell-to-cell interactions derived from CellPhoneDB. The names of the two most interconnected cell types are displayed (edge: identified cell-to-cell interaction; edge width: proportional to number of interactions between two cell types; node size: proportional to number of overall interactions). **(C)** Results of NicheNet analysis, in which the heatmap in the top left corner displays the z-normalized ligand activity scores (based on area under the precision recall curve (AUPR)) of the top 3 ligands for either the DE genes from C1Q+ macrophages or monocyte-like macrophages, respectively. On the right the top 250 interaction scores of the ligands’ target genes are colored by their interaction score. The heatmap at the bottom represents the mean expression (z-transformed by gene across all macrophage states; according to **Figure S5** **D**) of the ligands’ target genes in C1Q+ macrophages or monocyte-like macrophages from control and COPD. **(D)** The mean expression of the top 6 ligands in all identified BALF cell types for either COPD or control patients (z-transformed by gene) is displayed. **(E)** Box plot with marked median of the measured protein expression (by Olink Proteomics) in BALF of LAP TGF-β1 in COPD and control with representation of individual donors (control n = 11, COPD n = 12; error bars indicating the standard deviation; statistics based on Wilcoxon rank sum test). **(F)** Representation of inferred ligand-to-target signaling path for TGF-β1 derived from the NicheNet analysis. The nodes representing the genes are colored by the expression fold change between COPD and control patients. **(G)** Box plots (with marked median values) showing the mean expression per sample of TGF-β-signaling genes (error bars indicating the standard deviation; statistics are based on the Wilcoxon rank sum test). The underlying data are obtained from Shaykhiev et al. (17). signal., signaling; TF, transcription factor; MФ, macrophage; regulat., regulatory; mono, monocyte; DC, dendritic cell; transcript., transcriptional.

To define potential upstream regulators for changes observed in COPD, we focused on those macrophage states with a minimum of 30 DE genes between COPD and control (**Table S5**). Representation of predicted transcriptional regulators in an UpSet plot showed that *YY1*, which is an important modulator of TGF-β1 and NOTCH signaling, was the only predicted transcription factor (TF) shared by all macrophage states included in the analysis (**Figure 5A**). Elevated TGF-β signaling was further supported by the identification of the TFs *TFE3* and *MYOD1* with co-regulation being present in monocyte-like macrophages (cluster MФ5) and C1Q^+^ macrophages (cluster MФ3) which was similarly true for the NOTCH signaling related TFs *HES1* and *HEY1*. Other predicted signaling cascades included WNT signaling (*e.g. TCF3/4*, *MYC* and *NFATC1/3*) and TNF/NF-κB signaling (*e.g. CEBPB* and *REL*). These major pathways suggested that signals from the microenvironment are important drivers for transcriptional alterations in macrophages. We next applied CellPhoneDB, which models cell-to-cell communications based on known receptor-ligand interactions (19). Network construction of cell-to-cell interactions within control samples revealed monocyte-like and C1Q^+^ macrophages to be the major network hubs (**Figure 5B**). In COPD, cell-to-cell communication was increased, which was particularly obvious for C1Q^+^ and monocyte-like macrophages (**Figure 5B**). Among the predicted monocyte-like macrophage interactions, which showed the strongest difference between COPD and the control, we identified several receptor-ligand pairs associated with the TNF superfamily (**Figure S5A**). Furthermore, we found an increased likelihood of interaction between the ligand TGF-β1 and the receptor TGFBR1 in COPD.

To corroborate this model, we applied NicheNet (20) to monocyte-like and C1Q^+^ macrophages exhibiting the most cell-to-cell-interactions (**Figure 5B**) and most DE genes in COPD **(** **Figure 4B** **)**. Ligand activity analysis allowed selection of the top 3 ligands that best predicted DE genes in one of the two macrophage states (**Figure 5C**, **Figure S5B, C** **)**. *TGFB1*, *PTPRM* and *PSAP* were predicted to regulate monocyte-like cells, while C1Q^+^ macrophages were influenced mainly by *INHBA* and to a lesser extent *ADAM12*. As *INHBA* is part of the TGF-β superfamily and shares the same signaling cascade *via* SMAD2/3/4/7, there might be more commonality in ligand activity within these two macrophage states. Most of the genes, for which expression is predicted to be regulated by the aforementioned ligands, showed a clear DE pattern between COPD patients in both C1Q^+^ and monocyte-like macrophages **(** **Figure 5C** **)**, but only weak expression in the other macrophage states (**Figure S5D**). In contrast, visualization of the expression of the predicted ligands across the different immune cell types from BALF revealed no clear differences between COPD versus control cells for *INHBA*, *PSAP* and *ADAM12* **(** **Figure 5D** **)**. If these genes play a role in COPD, the major sources might be cells not present in BALF. For instance, Activin-A, whose subunit is encoded by *INHBA*, is known to be upregulated on lung epithelial cells from COPD patients (21). However, for the ligands *CSF1, PTPRM*, and *TGFB1*, we found a direct link between their ligand activity and the gene expression in BALF cells from COPD patients (**Figure 5D**). Since *TGFB1* was both predicted as a signaling pathway of transcriptional regulation (**Figure 5A**) and identified as a cell-to-cell-interaction partner for monocyte-like macrophages by CellPhoneDB (**Figure S5A**), we focused further analysis on this ligand. *TGFB1* is upregulated in COPD patients in eosinophils, C1Q^+^ macrophages, monocyte-like macrophages, neutrophils and mast cells (**Figure 5D**). To assess whether the increase in *TGFB1* expression is translated into elevated protein levels, we examined the BALF of COPD patients and control donors for the latency-associated peptide TGF-β1 (LAP TGF-β1), which serves as a surrogate for TGF-β1 protein levels. This analysis showed a tendency towards increased LAP TGF-β1 levels in COPD (**Figure 5E**), which is further supported by reports on elevated *TGFB1* levels in peripheral lung tissue from COPD patients (22).

In addition to elevated *TGFB1* expression in COPD, the receptors with the highest predicted interaction potential score for *TGFB1* (*TGFBR1* and *TGFBR2*) exhibited also higher expression in monocyte-like macrophages from COPD patients (**Figure 5F**). Further, we visualized NicheNet-predicted signaling and transcriptional regulation events between *TGFB1* and its putative target genes shown to be DE in COPD (**Figure 5F**). The nodes in the constructed path were colored according to the expression fold change between COPD and control. Among the transcriptional regulators were the classical TGF-β signaling mediators *SMAD3* and *SMAD4*, with *SMAD4* showing increased expression in COPD (**Figure 5F**).

Finally, further support for the importance of TGF-β signaling in COPD came from elevated expression of genes within the TGF-β signaling cascade in COPD patients but not smokers when compared to healthy non-smokers, as assessed in the dataset from Shaykhiev et al. (17) (**Figure 5G**). In summary, we predicted TGF-β signaling to be a prominent regulator of gene expression in BALF-derived macrophages in the context of COPD.

### The macrophage pool is supplied by blood monocytes in COPD

**Figure 6:**
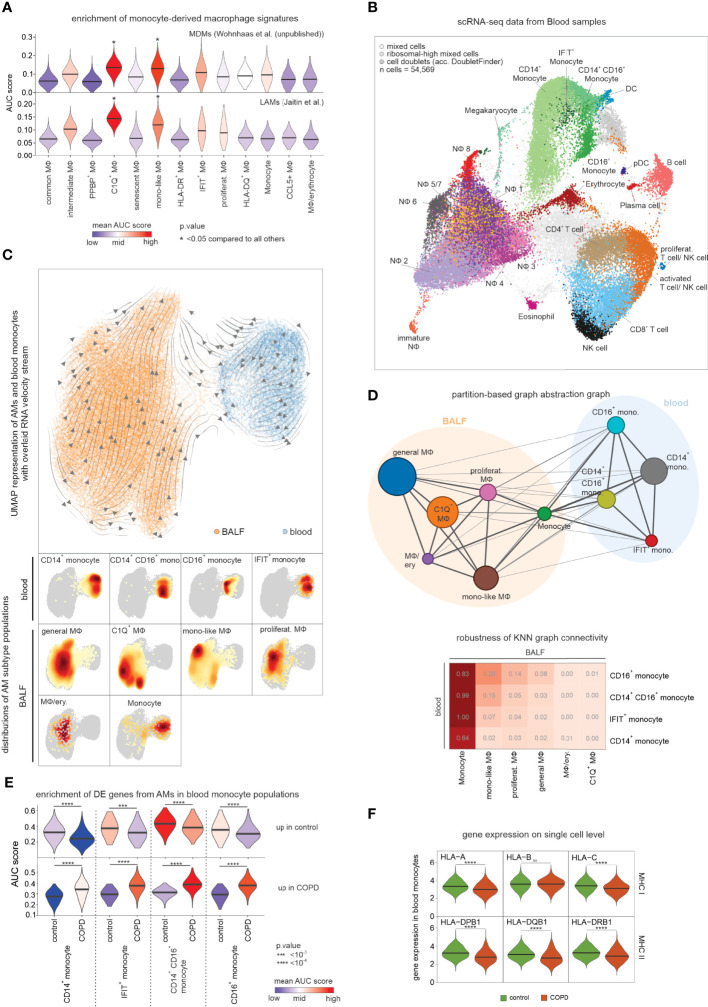
Assessing the relationship between blood monocytes and BALF macrophages. **(A)** Violin plots (with marked median values) displaying enrichment of human orthologues of murine monocyte-derived macrophage signature genes across macrophage states in COPD and control based on Area Under the Curve (AUC). **(B)** Integrated scRNA-seq data of blood immune cells annotated according to the four-step annotation approach (according to **Figure 1B**). **(C)** UMAP of embedded macrophages/monocytes from BALF and blood monocytes. Inferred main average vector flow is indicated by velocity streamlines that are projected as vectors. Locations of the main cell types (acc. to the combined labels from **Figure S6A**) in the UMAP are indicated by the heat maps at the bottom. **(D)** PAGA graph derived from embedded BALF and blood data (according to **Figure 6C**). The weight of an edge, which reflects a statistical measure of connectivity, is represented as the edge width. The table below summarizes the results of the PAGA connectivity calculation, where a value of 1 indicates a strong connection and 0 indicates a weak connection between two cell types. **(E)** Violin plots (with marked median values) displaying enrichment of macrophage-related DE genes (according to **Figures 4B, C**) in blood monocytes based on AUC. **(F)** Violin plots with marked median of the expression of HLA genes, in blood monocytes based on scRNA-seq data. The plots show the expression across the donors, whereby the donors were downsampled to the same number of cells, followed by downsampling to the same number of cells between COPD and control. The plots display cells with an expression > 0. BALF, bronchoalveolar lavage fluid; mono, monocyte; MФ, macrophage; NФ, neutrophil; proliferat., proliferating; MDM, monocyte-derived macrophage; LAM, lipid-associated macrophage; ns, means not significant.

We predicted TGF-β1 as an important regulator of monocyte-like macrophages (**Figure 5C**), and this cytokine has recently been identified as a crucial cytokine in macrophage differentiation (23). Tissue macrophage replenishment is linked to the local proliferation of tissue-resident cells, but also influx and subsequent differentiation of monocyte-derived cells from the circulation (24). The monocyte-like macrophages had transcriptional similarities to monocytes (**Figure 4B**) and, at the same time, their expression profile was regulated by typical signaling pathways of cell differentiation (**Figure 5A, C** **)**, suggesting that they may be derived from monocytes. To investigate whether the monocyte-like macrophage state represents an early stage of monocyte-to-macrophage differentiation, we used a gene signature of murine monocyte-derived macrophages (MDM) from the lungs of smoke-exposed mice (Wohnhaas *et al.*, *unpublished data*) and assessed the enrichment of orthologous genes in the human macrophage states (**Figure 6A**). The strongest enrichment of the MDM signature was found in monocyte-like (cluster MФ5) and C1Q^+^ macrophages (cluster MФ3). Utilizing orthologous gene signatures derived from murine lipid-associated macrophages (LAMs), which were shown to be monocyte-derived by lineage tracing (25), also revealed the strongest enrichment in monocyte-like and C1Q^+^ macrophages (**Figure 6A**). These enrichment analyses supported the hypothesis that monocyte-like, but also C1Q^+^ macrophages, were derived from monocytes.

To establish a direct link from circulating monocytes to the monocyte-related macrophages, we performed scRNA-seq of blood immune cells (**Figure 1**, **Figure 6B**) from the same donors from whom the scRNA-seq data of alveolar space immune cells were obtained. Application of the four-step cell type annotation approach (**Figure 1B**) identified the three known blood monocyte populations comprising classical monocytes (CD14^+^ monocytes), intermediate monocytes (CD14^+^CD16^+^ monocytes) and non-classical monocytes (CD16^+^ monocytes) along with a small monocyte population that expressed high numbers of interferon-associated genes (IFIT^+^ monocytes) (**Figure 6B**). We next described the relationship between blood-derived monocytes and alveolar space-derived monocytes and macrophages by building a model to determine which of the monocyte subtypes in the blood would most likely give rise to the monocyte-like macrophage state. For this purpose, we combined the blood and BALF data while considering donor batches. While this approach enabled the combination of the blood and alveolar space data, we observed a reduced resolution of the defined macrophage states and therefore continued with a simplified annotation for the analysis of the embedded data (**Figure 6C**, **Figure S6A**). Projection of RNA velocity vectors calculated by the scVelo method (26) in a batch-corrected manner onto the embedded data (**Figure S6B**) and inference of the main average vector flow visualized by velocity streamlines (**Figure 6C**) revealed a clear motion of blood monocytes towards the macrophages, further supporting circulating monocytes to be precursors of macrophages in the alveolar space. Since RNA velocity visualization on the UMAP did not reveal a clear link between individual macrophage states and blood monocyte subsets, we calculated a higher-order representation using partition-based graph abstraction analysis (PAGA) (27) (**Figure 6D**). The strongest connection was derived between blood monocytes and monocytes identified in the alveolar space. To evaluate the connectivity of the PAGA network more precisely, we used the connectivity matrix as a test statistic to define the highest likelihood for each of the blood monocyte subtypes to be related to the different macrophage states in the alveolar space (**Figure 6D**). The monocytes within the alveolar space served as positive controls indicating very high relationships. However, among the macrophage states, we could establish the strongest connections between the CD16^+^ monocyte subtype in blood and the monocyte-like macrophages in the alveolar space, further supporting that the monocyte-like macrophages are most likely an early functional state of macrophages after circulating monocytes enter this tissue compartment.

Lastly, we investigated whether the DE genes in macrophages from COPD patients (**Figure 4B, C** **)** were already altered in blood monocytes. For this purpose, we used the DE genes as signatures of up- and downregulated genes. Clearly, these signatures were altered in the different blood monocyte subtypes derived from COPD patients with CD14^+^CD16^+^ and CD16^+^ monocyte subtypes showing the strongest enrichment of macrophage DE genes upregulated in COPD (**Figure 6E**). Of particular interest, MHC class I and II genes were found to be expressed at lower levels in COPD-derived monocytes, supporting a systemic component of COPD leading to transcriptional changes in circulating monocytes (**Figure 6F**).

In summary, we provide evidence that blood monocytes contribute to the macrophage pool, with monocyte-like macrophages providing a link between blood and lung. The monocyte-like macrophages are elevated in the alveolar space of COPD patients (**Figure 2C**), suggesting an increased infiltration of blood monocytes. In addition, blood monocytes already show transcriptional changes reminiscent of those observed in cells from the alveolar space strongly arguing for a systemic component in COPD.

## Discussion

COPD is an inflammatory lung disease with a high global burden, increasing incidence, prevalence, morbidity and mortality, mainly due to rising air pollution and high smoking rates worldwide (2). Yet, the cellular and molecular mechanisms of this heterogeneous disease are far from being fully understood. Not surprisingly, the diagnosis of COPD is solely based on clinical parameters due to the lack of molecularly defined biomarkers and, as a consequence, causal therapies are lacking because of an incomplete understanding of the complex pathophysiology.

**Figure 7:**
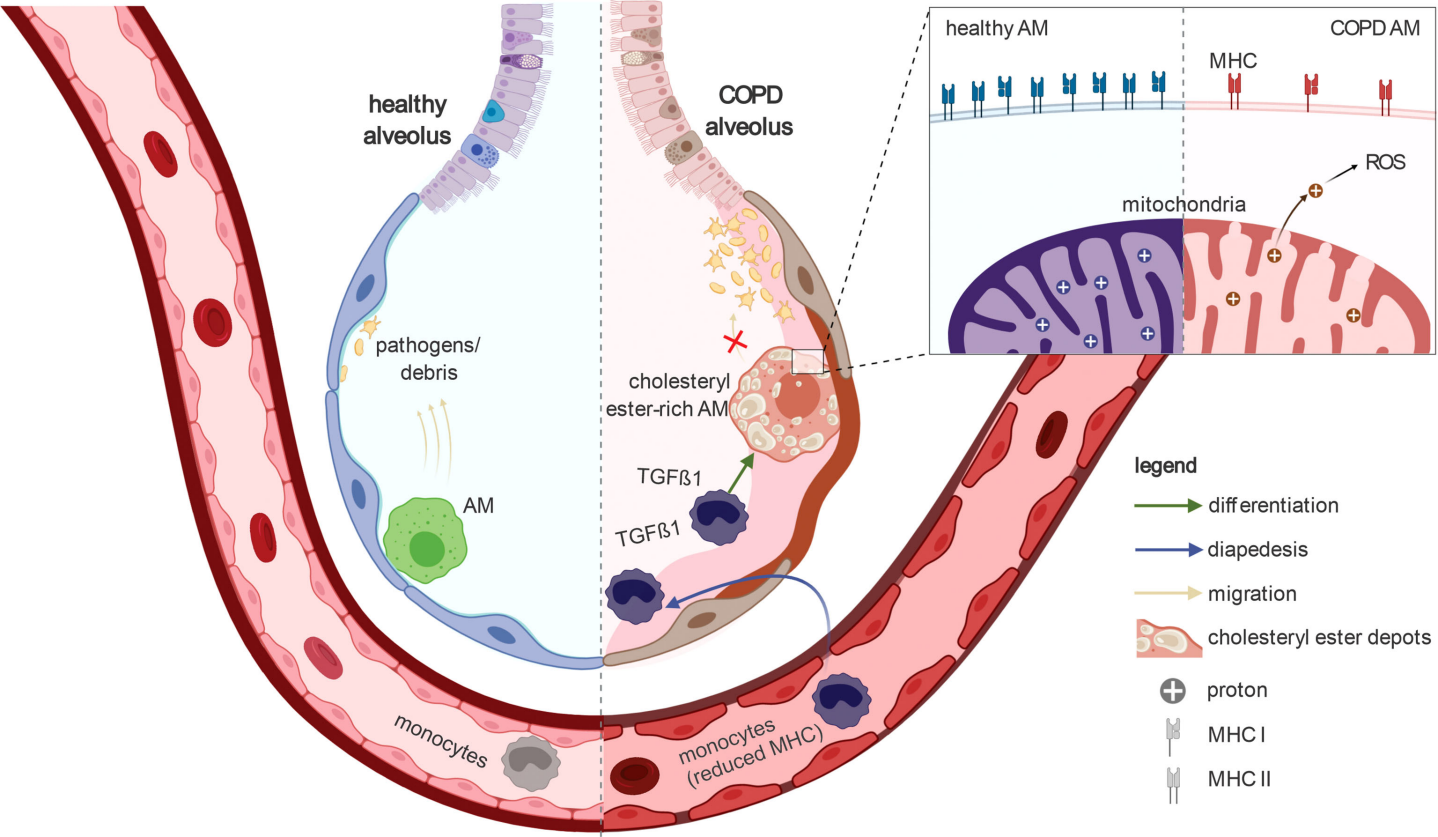
Schematic representation of the key findings of the present study In healthy lungs, alveolar macrophages survey the alveoli and remove pathogens and debris to enable proper gas exchange. In the alveoli of COPD patients, the alveolar macrophages accumulate cholesteryl esters. In addition, blood monocytes invade the alveoli and differentiate into alveolar macrophages. The transcriptome of COPD alveolar macrophages indicate TGF-β1-associated cell signaling especially in the early stages of monocyte-to-macrophage differentiation. The alveolar macrophages in COPD show a reduced ability to migrate towards chemokine. Furthermore, they express fewer MHC molecules; especially MHC class I. Together with the reduced phagocytosis of alveolar macrophages in COPD, the ability of these cells for immune surveillance is severely limited during the disease. In addition, their mitochondria are leaking (e.g. to protons) and therefore produce high amounts of reactive oxygen species. Taken together, the guardians of normal lung function (alveolar macrophages) are severely altered in COPD, preventing them from fulfilling their important physiological functions properly. Furthermore, the observation of reduced MHC expression in blood monocytes indicates that the manifestation of COPD has a strong systemic component.

Here, we characterized COPD-associated changes in immune cells from BALF and blood using scRNA-seq in combination with the application of advanced computational approaches. Focusing on alveolar macrophages, the most prevalent cell compartment in BALF, we found specific alterations in lipid metabolism, reduced expression of MHC class I molecules, and identified TGF-β1 as a major factor responsible for transcriptional reprogramming in COPD. Overall, our results indicate stressed and dysfunctional macrophages in COPD. Changes of the molecular phenotype were further supported by functional analysis, illustrating mitochondrial leakage and reduced chemotaxis. In addition, proliferating and monocyte-like macrophages were elevated in COPD, with evidence that the latter were derived from blood monocytes (**Figure 7**).

Recently, it has been hypothesized that reprogramming of disease-related cells as a potential therapeutic option might only be possible at earlier stages (28). We therefore focused on patients diagnosed with early clinical stage disease (GOLD stage 2). Single-cell transcriptomes from BALF showed many different cellular states within the myeloid compartment, both in COPD patients and controls. We identified numerous alterations, both cell state-specific but also myeloid compartment-wide changes between COPD and controls. Of particular interest is the identification of reduced expression of MHC class I molecules across macrophage states in COPD. This finding is in accordance with previous studies linking downregulation of surface MHC class I in COPD with impaired immunoproteasome activity (29). Macrophages expressing low-level MHC class I are less efficient in inducing antiviral immune responses, which may explain the high susceptibility of COPD patients to viral infections, one of the main reasons for disease exacerbations.

To understand the regulation of the DE genes, we performed transcription factor binding prediction, receptor-ligand interaction modeling, and downstream transcriptional signature prediction, which indicated TGF-β signaling followed by NOTCH-, WNT-, and TNF-signaling to be elevated in COPD. The predicted pathways might also be involved in immunosenescence in COPD. For example, TGF-β1 can signal *via* the mTOR pathway, which was recently associated with cellular senescence in lung cells (30). As COPD develops preferentially in elderly people who often suffer from several comorbidities, cellular aging has been suggested as a hallmark of the disease (31). Features of cellular senescence comprise an increase in the number of mitochondria and mitochondrial dysfunction, which is reflected by increased proton leakage and an associated increase in reactive oxygen species (ROS) production (16). Oxidative stress due to increased ROS production is a feature of COPD and there is evidence that this is partly due to mitochondrial dysfunction (32). In line with cellular senescence, we found increased proton leakage in mitochondria of macrophages from COPD patients. Additionally, the reduced chemotactic capacity of macrophages in COPD might also be a result of aged immune cells (33). Reduced migratory capacity of macrophages can have deleterious consequences for the lung, as it reduces the efficient removal of pollutants from the alveolar space, which can lead to cell death and the induction of inflammation. Moreover, the clearance of the alveolar space is further deteriorated due to decreased phagocytosis in macrophages in COPD (34).

TGF-β signaling can induce downregulation of MHC expression. This effect has been associated with signaling *via* SMAD4 (35), which gives a direct link between the predicted intercellular signaling pathways and the DE genes observed between COPD and control patients. Moreover, TGF-β1 is a known inducer of ADAM10 and ADAM17 expression (36, 37) and is described to be essential for macrophage homeostasis and the differentiation of monocytes into macrophages (23). Following up on this idea, we performed PAGA and RNA velocity analysis of cells from the peripheral blood and the alveolar space. This model suggested that a proportion of the macrophage pool is replenished from the systemic monocyte pool circulating in peripheral blood. A recently proposed model (24) suggested that under homeostatic conditions survival of tissue-resident macrophages is supported by self-renewal within the local microenvironment while monocyte recruitment is rather limited. During inflammation, tissue-resident macrophages retain the ability to self-renew, but at the same time many blood-derived monocytes are recruited (38). In COPD, we found evidence for both, local proliferation of some macrophages and recruitment of blood monocytes. Indeed, monocyte-like and C1Q^+^ macrophages exhibited strong enrichment of monocyte-derived macrophage signatures (25). Further, RNA velocity analysis supported a differentiation process from blood monocytes, particularly the CD16^+^ subset towards the monocyte-like cell state within the alveolar macrophage compartment, which is in line with previous findings demonstrating that the murine counterpart of human CD16^+^ monocytes can differentiate into lung macrophages (39).

### Limitations of this study

The analysis of high quality-biosamples is an important prerequisite for high-resolution analysis such as single cell transcriptomes. While we screened many more patients, only a subfraction of BALF samples was of sufficiently high quality for further analyses. In addition, since the beginning of the pandemic we were not able to obtain further BALF samples due to hospital restrictions. While the study was comparable in size to many single cell transcriptome studies prior the pandemic, the last two years have seen an explosion of larger studies, in particular related to COVID-19. As a consequence, the size of the study now appears rather small. Yet, we have identified several important biological findings that characterize early-stage COPD. We anticipate that our study will provide a framework for further functional studies on the immune compartment in COPD, with a particular emphasis on metabolism, but also to further understand the patient heterogeneity we observed for some of the functional outcomes. In this context, it might be of particular interest that the blood compartment also showed already alterations that might be more easily assessed in future studies due to the easier access to this tissue compartment.

We clearly could show that COPD-related signatures derived from BALF-derived macrophages were already enriched in the peripheral blood monocyte pool, particularly in CD14^-^CD16^+^ and CD14^+^CD16^+^ subsets. These findings indicate that the pathophysiology of COPD is not restricted to the lung. More specifically, reduced MHC expression was also observed on circulating blood monocytes, which further underlines a systemic component of COPD (40). Importantly, elevated levels of TGFB1 have been described in plasma of COPD patients (41) that could explain the low MHC expression in blood monocytes. Finally, as we provide all single-cell transcriptome data and analyses in an integrated fashion on https://www.fastgenomics.org/ (**Figure S7**) our data are easily accessible for further analysis.

## Methods

### Contact for reagent and resource sharing

Further information and requests for resources and reagents should be directed to and will be fulfilled by the lead contact, Prof. Dr. Joachim L. Schultze (joachim.schultze@dzne.de). A detailed list of the used reagents and resources is provided in **Table S7**.

## Subject and method details

### Human specimens

Human studies were approved by the ethics committees of the University of Bonn and University hospital Bonn (local ethics vote 076/16). All patients provided written informed consent according to the Declaration of Helsinki before specimens were collected. Each individual included in this study was diagnosed and the disease stage was stratified according to the recommendations of the global initiative for chronic obstructive lung disease (GOLD) (COPD recommendations, 2020), with a ratio of post-bronchodilator (salbutamol 400 µg) forced expiratory volume in 1 s (FEV1) to forced vital capacity (FVC) of less than 0.7, and moderate airflow limitation (50% <= FEV1 < 80%). For scRNA-seq, the eligible patients were aged 40 years or older and were either current or ex-smokers. Since COPD has recently been suggested to be a clinical syndrome rather than a single disease (42), we anticipated that despite the focus on GOLD 2 patients, the current study should include a spectrum of COPD patients (**Table S1**). For example, the generated dataset comprised COPD GOLD 2 patients with different emphysema proportions, exacerbation histories and even a patient suffering from combined pulmonary fibrosis and emphysema (CPFE). The latter patient was admitted based on an external diagnosis of COPD that was later diagnosed as CPFE. This disease type was first described by Cottin et al. (43) and is defined radiologically by the presence of classical features of emphysema in the upper lobes and pulmonary fibrosis in the lower lobes and subnormal lung volumes and severe reduction of CO transfer. Irrespective of the expected heterogeneity within the COPD GOLD 2 patient cohort, stringent exclusion criteria for the current study were a primary diagnosis of asthma with a physician-judged need for oral corticosteroid therapy, clinically significant cardiovascular disorders or laboratory abnormalities and unstable concurrent disease (e.g. exacerbation of disease) that could have affected safety (as judged by the investigator). Individuals suffering from chronic cough without any signs of severe lung pathophysiology or subnormal lung functions served as control donors.

### Isolation of cells from bronchoalveolar lavage fluid

Human BALF was obtained from patients with or without COPD *via* bronchoscopy (at the University hospital Bonn). BALF was performed according to the official American Thoracic Society guideline for interstitial lung disease patients to ensure highest quality of biospecimen material (44). According to these guidelines, we excluded more than half of the clinical samples from further analyses because either the volume of saline solution recovered compared to the amount previously injected into the lungs during bronchoscopy was too low, or blood contamination or increased upper respiratory secretion was present. Each of these factors has an influence on the differential cell count of BALF samples and would have therefore had a negative effect on the analysis results. BALF samples fulfilling the quality criteria were once washed with PBS supplemented with 1 mM EDTA followed by washing with PBS supplemented with 2% fetal calf serum (FCS) and 1 mM EDTA. Throughout the isolation process, the samples were kept at 4°C and centrifugation steps performed at 300 g for 10 min. To exclude any macroscopic non-cellular particles and non-immune cells from further analyses, immune cells were enriched with MACS columns by using CD45 microbeads according to manufacturer’s instructions.

### Isolation of peripheral blood mononuclear cells and granulocytes

For the assessment of relationship analysis of the myeloid cell compartment in BALF with cells from the systemic circulation, we obtained venipuncture blood on the day of bronchoscopy. PBMC were obtained by Pancoll density centrifugation (at 20°C and 700 g for 25min with centrifugation break was turned off) of the peripheral blood. After harvesting PBMC from the interphase, all further steps were conducted at 4°C. Granulocytes were recovered from the granulocyte/erythrocyte fraction using cold ACK (ammonium chloride potassium) lysing buffer (1.5M NH_4_Cl, 0.1M KHCO_3_ and 1mM EDTA in H_2_O with pH 7.4 at 8°C) to lyse erythrocytes, followed by a washing step with PBS supplemented with 2% FCS and 1 mM EDTA. All centrifugation steps required for granulocyte isolation were performed with max. 300 g for 10 min. To assess the granulocyte fraction in further analyses (particularly in scRNA-seq experiments, **Table S1**), it was mixed with the PBMC fraction in the ratio PBMC:granulocytes = 2:1. Finally, the PBMC/granulocyte mix was stained with CD45 microbeads for 15 min in order to use a magnetic field in the cell loading of Seq-Well arrays (see below). This artificial ratio allowed to assess the granulocytes in addition to the PBMCs without sequencing the majority of blood immune cells being granulocytes allowing sufficient granularity in the PBMC fraction.

### Flow cytometric data generation

Cells were resuspended in PBS supplemented with 2% FCS and 1 mM EDTA for surface marker staining (**Table S2**). To distinguish live from dead cells, the cells were incubated with LIVE/DEAD Fixable Yellow Dead Cell Stain Kit (1:1000) at room temperature for 15 min protected from light. After washing, human FcR blocking reagent was included to reduce unspecific staining (incubation on ice for 15 min). Next, surface antibodies were added and after 30 min incubation at 4°C in the dark, cells were washed and analyzed either on BD FACSAria III (Becton Dickinson; 3 lasers: violet, blue, and red) for acquisition and sorting or on BD FACSCanto II (Becton Dickinson; 2 lasers: blue and red) for acquisition only (**Table S2**). Fluorescence-minus-one (FMO) controls were prepared for non-lineage markers.

### Flow cytometric data analysis

Preliminary data analysis was performed using FlowJo software (version 10). The package ‘flowCore’ (version 1.46.2 (45)) was used to import the compensated data into R. For dimensionality reduction with UMAP implementation in R (version 0.2.1.0 (46)), fluorescence parameters were transformed with logicleTransform (47, 48). Subsequent clustering of the dataset was performed with the PhenoGraph algorithm implemented in the ‘Rphenograph’ package (version 0.99.1 (49)) by setting the number of nearest neighbors to 25. Based on marker detection, the major cell types in the BALF were defined as macrophages (Lin^-^ (including CD3, CD19 and CD56) CD66b^-^ HLA-DR^+^ autofluorescence^+^), monocytes/DCs (Lin^-^ CD66b^-^ autofluorescence^-^ HLA-DR^+^ and either CD14^+^, CD16^+^ or CD14^+^ CD16^+^), granulocytes (Lin^-^ HLA-DR^low^ autofluorescence^low^ CD66b^+^ and either CD16^-^ Siglec-8^+^, CD16^+^ Siglec-8^+^ and CD16^+^ Siglec-8^-^) and T cells/NK cells including a small fraction of B cells (autofluorescence^-^ CD14^-^ CD66b^-^ Lin^+^ and further resolved using the lymphoid panel (**Table S2**)). In blood, the major cell types were defined as monocytes/DCs (CD3^-^ CD19^-^ CD56^-^ CD66b^-^ HLA-DR^+^ and either CD14^+^, CD16^+^ or CD14^+^ CD16^+^), T cells/NK cells (CD14^-^ CD33^-^ CD66b^-^ CD11c^-^ CD123^-^ CD19^-^ and either CD3^+^ CD4^+^, CD3^+^ CD8^+^ or CD56^+^), granulocytes (CD3^-^ CD19^-^ CD56^-^ HLA-DR^low^ CD66b^+^ CD16^+^ and either CD16^-^ Siglec-8^+^, CD16^+^ Siglec-8^+^ and CD16^+^ Siglec-8^-^) and B cells (CD14^-^ CD33^-^ CD66b^-^ CD11c^-^ CD123^-^ CD3^-^ CD56^-^ CD19^+^). According to these marker combinations, the identified clusters were annotated. To unify and simplify the analysis across multiple datasets, an annotated dataset was defined as the reference and the other flow cytometry datasets were projected onto its UMAP coordinates using the ‘umap’ object of the reference dataset and the logicle transformed flow cytometry data of the second dataset as input for the predict function in R. In addition, the same function was also used to predict the clusters of the remaining datasets with respect to the reference dataset. This step, together with the visualization of detected markers, made it possible to assess both the accuracy of the projection method and the cell type annotation of the projected datasets.

We performed differential marker intensity measurements across individuals based on the Cohen’s d definition of effect size as follows:


$$effect\;size=\;\frac{Mean_{complete\;stain}-\;Mean_{FMO}\;}{\sqrt{\frac{SD_{complete\;stain}^{2}+SD_{FMO}^{2}\;}{2}}}$$

with FMO = fluorescence minus one and SD = standard deviation. This procedure was followed since we observed strong variability in autofluorescence intensities of macrophages among donors, despite strictest standard operating procedure (SOP) compliance and the use of SOPs for application settings (50) during flow cytometry to minimize potential biases that can occur during sample-to-sample flow cytometry comparisons.

### MitoStress assay on seahorse

For the analysis of the metabolic state of donor-derived alveolar macrophages, freshly obtained BALF was centrifuged for 10 min at 300 g. Cell pellet was then washed carefully in PBS (supplemented with 0.02% EDTA) and finally resuspended in MACS buffer. Cell suspension was then stained for 15 min with CD66b microbeads and depleted from granulocytes according to manufacturer instructions. Granulocyte-depleted cell suspension was counted and seeded in Seahorse XF RPMI medium (supplemented with 2 mM L-glutamine, 1 mM sodium pyruvate, 10 mM glucose, adjusted to pH 7.4 prior to the assay) at a concentration of 200,000 cell per well; for each sample, 2 to 4 technical replicates were performed. Cells were then incubated for 30 min in a 37°C incubator, washed two times with pre-warmed Seahorse XF RPMI medium to remove all non-adherent cells and loaded onto the Seahorse XFe96 Analyzer (Agilent). After 3 cycles of baseline measurement, whereby one cycle is defined as 3 min of initial mixing and 3 min measurement, the cells were subsequently injected with Oligomycin (1:1000), FCCP (1:500) and finally a combination of Antimycin A and Rotenone (both 1:2000). Following each injection, oxygen consumption rate (OCR) was measured for 3 cycles.

After the assay, the relative cellular number was determined *via* crystal violet staining. Shortly, cells were fixed with 4% PFA for 5 min at room temperature and stained for 30 min with crystal violet (0.05% in H_2_O). After two washes with H_2_O the staining was air dried and the formed crystals were dissolved in 200 µL of methanol. Absorbance at 590 nm was measured and used to normalize the Seahorse assay within the Wave software (Agilent). The normalized data were finally exported, further analyzed and visualized in R, with values adjusted to the measured baseline (baseline-corrected). Basal respiration was calculated as baseline OCR at the beginning of the measurement – (OCR after addition of rotenone + antimycin A), maximal respiration as OCR after addition of FCCP – (OCR after addition of rotenone + antimycin A), and proton leak as OCR after addition of oligomycin – (OCR after addition of rotenone + antimycin A) according to schema in **Figure S3E**.

### Migration assay

Migration was analyzed in 24-well transwell plate containing a 8 µm polycarbonate membrane. Macrophages were first purified by FACS according to the expression of CD45, CD66b, HLA-DR and the absence of CD3, CD19 and CD56. Cells were also selected according to the strong autofluorescence signal (51). Macrophages were cultured in 300 µL starvation medium (RPMI 1640 medium supplemented with 0.5% FCS and 1% penicillin/streptomycin) and 50,000 macrophages were seeded in each upper well, while the lower chamber was filled with 700 µL starvation medium only. After an incubation of 1 h in a 37°C incubator, the medium in the upper chamber was exchanged with 300 µL fresh starvation medium and the medium in the lower chamber with 700 µL starvation medium supplemented with 100 ng/mL recombinant human CCL3. The seeded macrophages were incubated at 37°C overnight. Next, cells on the upper filter surface were removed with a cotton swab. Transmigrated cells on lower filter surface were incubated with 2 µM CFSE in 700 μL PBS for 10 min in a 37°C incubator. The transwell inserts were then transferred into wells containing 700 µL RPMI 1640 medium supplemented with 10% FCS and 1% penicillin/streptomycin and incubated for 10 min in a 37°C incubator. Finally, transwell inserts were washed with PBS and imaging of cells was performed using an inverted fluorescent microscope (Nikon) with a 10-fold objective and GFP filter. The number of migrated cells was quantified using ImageJ [version 2 (52)].

### Measurement of proteins in BALF

After isolation of cells (see above), the supernatant of BALF samples of both COPD patients and controls were collected and frozen at −80°C before proteomics measurement. Protein levels from cell-free BALF samples were determined using the INFLAMMATION panel from Olink Proteomics, a commercial multiplex immunoassay for high-throughput detection of 92 inflammation-related protein biomarkers. The obtained normalized results (**Table S4**) were further analyzed in R, whereby proteins were kept for visualization that showed a statistically significant difference (Wilcoxon rank sum test-based p-value < 0.1) between COPD and control samples.

### Lipidomics of macrophages in BALF

Macrophages were sorted, washed with PBS and with 150 mM ammonium acetate in a glass tube, pelleted (300 g with slow brake), and frozen at -80°C until analysis. To the pellet, 500 µL of extraction mix (CHCl_3_/MeOH 1/5 containing internal standards: 210 pmol PE(31:1), 396 pmol PC(31:1), 98 pmol PS(31:1), 84 pmol PI(34:0), 56 pmol PA(31:1), 51 pmol PG (28:0), 28 pmol CL(56:0), 39 pmol LPA (17:0), 35 pmol LPC(17:1), 38 pmol LPE (17:1), 32 pmol Cer(17:0), 99 pmol SM(17:0),55 pmol GlcCer(12:0), 14 pmol GM3 (18:0-D3), 359 pmol TG(47:1), 111 pmol CE(17:1), 64 pmol DG(31:1), 103 pmol MG(17:1), 724 pmol Chol(d6), 45 pmol Car(15:0)) were added and each sample sonicated for 2 min followed by centrifugation at 20,000 g for 2 min. The supernatant was collected into a new tube and 200 µL chloroform and 800 µL 1% AcOH in H_2_O were added. The sample was then briefly shaken and spun for 2 min at 20,000 g for 2 min. 200 µL chloroform and 800 µL 1% AcOH in H_2_O were added to the supernatant, briefly shaken and spun for 2 min at 20,000 g. The lower phase was transferred into a new tube and evaporated in a speed vac (45°C, 10 min). Spray buffer (500 µL of 8/5/1 2-propanol/MeOH/H_2_O, 10 mM ammonium acetate) was added, sonicated for 5 min and infused at 10 µL/min into a Thermo Q Exactive Plus spectrometer (Thermo Fisher Scientific) equipped with the HESI II ion source for shotgun lipidomics. MS1 spectra (res. 280000) were recorded in 100 m/z windows from 200 – 1200 m/z (pos.) and 200 – 1700 m/z (neg.) followed by recording MS/MS spectra (res. 70000) by data independent acquisition in 1 m/z windows from 200 – 1200 (pos.) and 200 – 1700 (neg.) m/z.

Raw files were converted to mzml files and imported into and analyzed by LipidXplorer (version 1.2.8 (53)) software using custom mfql files to identify sample lipids and internal standards. For further data processing, absolute amounts were calculated using the internal standard intensities followed by normalization of the identified lipids on total lipid content. Lipid class sums were calculated for each donor and log_2_-transformed. Differential lipid classes were calculated between COPD GOLD 2 vs control samples using the ‘limma’ package [version 3.42.2 (54)] under consideration of ‘date of sampling’.

### Nanodroplet-based scRNA-seq

For comparison of nanodroplet-based scRNA-seq with array-based scRNA-seq (Seq-Well technology, see below), cell preparations derived from three blood and three BALF donors were split in half to be further processed with the two different scRNA-seq technologies by two teams simultaneously. For each donor, 10,000 BALF or blood-derived cells were loaded onto the Chromium™ Controller instrument (10x Genomics) using the Chromium™ Single Cell A Chip Kit together with the Chromium™ Gel Bead Kit v2 following the manufacturer’s recommendations. Libraries were prepared using Chromium™ Single Cell 3’ Library Kit v2 according to manufacturer’s recommendations and sequenced paired-end as followed: Read 1 26 cycles, i7 index 8 cycles and Read 2 56 cycles on a NextSeq500 instrument (Illumina) using High Output v2.1 chemistry. Single-cell data was demultiplexed and converted into fastq format using bcl2fastq2 (v2.20).

### Preparation of Seq-Well arrays

Seq-Well arrays were prepared as described by Gierahn et al. (6). Briefly, Sylgard base and crosslinker were mixed at 10:1 ratio for 10 min, placed under vacuum pressure for 15 min to remove air bubbles and were next poured for a 2 h incubation at 70°C into a wafer with a mounted 86,000 well pattern-holding microscope slide. The arrays were then removed from the molds, excess silicone was cut off with a blade and were prepared for the functionalization process. This protocol adds chemical moieties to the surface of the arrays which facilitate the sealing of a semi-permeable polycarbonate membrane and the interchange of lysis and RNA hybridization buffers. Arrays were rinsed with EtOH, plasma treated for 10 min and successively submerged in APTES (0.05% APTES in 95% EtOH), acetone and PDITC buffers (0.2% PDITC, 10% pyridine, 90% DMF). Upon further washes with acetone, the arrays were spun and dried at 70°C for 2 h. Among the most critical steps in the protocol was the incubation of the arrays with 0.2% chitosan solution (pH=6.3) at 37°C for 1.5 h, after which an overnight incubation in PGA buffer (20 µg/mL polyglutamic acid, 2 M NaCl, 100 mM sodium carbonate (pH=10)) at room temperature under vacuum pressure followed. Finally, the arrays were removed from the vacuum and were rotated for 3 h at room temperature and subsequently moved to 4°C for at least 24 h before use.

### Preparation of Seq-Well libraries and sequencing

Seq-Well libraries were generated as recently described by Gierahn et al. (6). After loading of the functionalized arrays with mRNA capture beads, 20,000 CD45^+^ cells were applied that were previously coated with CD45^+^ magnetic beads (see above) and suspended in RPMI 1640 medium supplemented with 10% FCS. During the incubation time of 10 min, the loaded arrays were placed on a strong magnetic plate to support the settling of the cells *via* a magnetic field. After repetitive washing with PBS and soaking with RPMI 1640 medium, the arrays were sealed using polycarbonate membranes that were 7 min treated with air plasma under mild vacuum (Diener electronic). Following a 30 min incubation time in a 37°C cell culture incubator, the arrays were incubated in lysis buffer (5M guanidine thiocyanate, 1mM EDTA, 0.5% Sarkosyl and 1% β-mercaptoethanol in H_2_O) for 20 min and then placed in hybridization buffer (2M NaCl, 3mM MgCl_2_ and 0.5% Tween-20 in PBS) for 40 min. Next, the mRNA capture beads were washed from the arrays and collected using washing buffer (2M NaCl, 3mM MgCl_2_ and 20mM Tris-HCl pH 8.0 in H_2_O). The reverse transcription was performed on the bead pellet using a Maxima Reverse Transcriptase reaction (Maxima RT buffer, 4% Ficoll PM-400, 1mM dNTPs, 1U/µL RNase inhibitor, 2.5 µM template switch oligonucleotide (TSO) primer and 10U/µL Maxima Reverse Transcriptase in H_2_O) for 30 min at room temperature followed by 90 min incubation at 52°C with end-over-end rotation. The reaction was stopped by washing the beads with TE buffer (10mM Tris-HCl pH 8.0 and 1mM EDTA in H_2_O) supplemented with 0.1% Tween-20 (TE-TW) and TE buffer supplemented with 0.5% SDS (TE-SDS). After a washing step in 10mM TrisHCl pH 8.0, excess primers were digested in an exonuclease reaction (ExoI buffer and 1U/µL ExoI in H_2_O) for 50 min at 37°C with end-over-end rotation and washed in TE-TW and TE-SDS. Beads were resuspended in 500 µL H_2_O and counted with a Fuchs-Rosenthal cytometer in bead counting solution (10% PEG, 2.5 M NaCl). Pools of 5,000 beads (10 µL) were then added to 40 µL PCR reactions (2X KAPA HiFi Hotstart Readymix and 25 µM SMART PCR primer in H_2_O) for the amplification of reverse transcribed cDNA libraries (95°C for 3 min, 4 cycles of 98°C for 20 s, 65°C for 45 s, 72°C for 3 min, 12 cycles of 98°C for 20 s, 67°C for 20 s, 72°C for 3 min and final extension of 72°C for 5 min). After PCR, 16,000-20,000 beads were combined (thereafter referred to as ‘pools’) and further processed. The pools were cleaned with 0.6x volumetric ratio AMPure XP beads (5 min incubation with beads, followed by 3 min on the magnet, two washes with 80% EtOH, 5 min dry-out, elution with 13 µL H_2_O for 3 min, followed by 2 min on the magnet for collection of the eluent) and the library integrity was assessed using a High Sensitivity D5000 assay for the Tapestation 4200 (Agilent).

To reduce library costs, we produced homemade Tn5 transposase according to Picelli et al. (55). Briefly, the Tn5 coding sequence (tnpA gene from *Escherichia coli*, Uniprot accession number: Q46731, residues 1-476) was purchased as a synthesized gene containing the mutations E54K and L372P for hyperactivation of the enzyme. Overhangs with the restriction sites *Xba*I and *Spe*I were used for cloning into pTXB1 vector, generating a Tn5-Intein-CBD fusion construct. The Tn5 coding sequence was validated by Sanger sequencing. Next, the pTXB1-Tn5-Mxe-CBD plasmid was transformed into the *E.coli* strain BL21. Cells were grown in LB media supplemented with ampicillin at 37°C to an OD_600_ 0.8. The temperature was then lowered to 10°C and protein expression was induced by addition of 0.25 mM IPTG. After incubation at 23°C for 4 h cells were harvested by centrifugation at 15,000 rpm on a JA 25.50 rotor (Beckman) for 20 min at 10°C. The cell pellet was resuspended in running buffer (20 mM Hepes-KOH, 0.8 M NaCl, 1 mM EDTA, 10% glycerol, 0.2% Triton-X 100) supplemented with 1 mM PMSF and disrupted by sonication. After centrifugation of cell debris at 15,000 rpm on a JA 25.50 rotor (Beckman) for 30 min at 10°C, residual nucleic acid contaminations from *E.coli* were precipitated by dropwise addition of polyethyleneimine pH 7.5 to a final concentration of 0.3%. The lysate was cleared by centrifugation at 12,000 rpm on a JA 25.50 rotor (Beckman) for 10 min at 4°C. Chitin resin (10 mL) was equilibrated with running buffer and then incubated with the prepared lysate for 1 h at 4°C. Beads were washed with 10 column volumes of running buffer. For elution by self-cleavage *via* the intein-tag, the Tn5-loaded resin was incubated overnight at 4°C in 3 mL elution buffer (20 mM Hepes-KOH, 0.8 M NaCl, 1 mM EDTA, 10% glycerol, 0.2% Triton-X 100, 100 mM DTT), followed by dialysis at 4°C overnight in dialysis buffer (100 mM Hepes-KOH, 0.2 M NaCl, 0.2 mM EDTA, 2 mM DTT, 0.2% Triton-X 100, 20% glycerol). The protein concentration was determined using Bradford Assay. Glycerol was added to a final concentration of 50% to the protein sample.

To load Tn5 with linker oligonucleotides (Tn5ME-B/Tn5MErev (Tn5ME-B: 5`- TCTCGTGGGCTCGGAGATGTGTATAAGAGACAG-3`; Tn5MErev: 5`-[phos]CTGTCTCTTATACACATCT-3`);), single-stranded oligonucleotides were mixed in a 1:1 ratio. For pre-annealing, 2 µL of the oligonucleotide solution was mixed with 8 µL of H_2_O and incubated in a thermocycler (95°C for 3 min, 70°C for 3 min and 45 cycles of temperature reduction (-1°C per 30 s)). The annealed oligonucleotides (0.25 vol.) were added to 0.1 vol. Tn5 solution and supplemented with •0.4 vol. glycerol (100%), 0.12 vol. dialysis buffer and 0.13 vol. H_2_O. After incubation for 60 min at room temperature, the protein was stored at -20°C.

The cDNA libraries (1 ng) were tagmented with the prepared single-loaded Tn5 transposase in TAPS-DMF buffer (50mM TAPS-NaOH (pH 8.5), 25mM MgCl_2_, 50% DMF in H_2_O) for 10 min at 55°C and the tagmented products were cleaned with the MinElute PCR kit following the manufacturer’s instructions. Finally, a master mix was prepared (2X NEBNext High Fidelity PCR Master Mix, 2.5 µM barcoded index primer, 2.5 µM P5-SMART-PCR primer) and added to the samples to attach the Illumina indices to the tagmented products in a PCR reaction (72°C for 5 min, 98°C for 30 s, 15 cycles of 98°C for 10 s, 63°C for 30 s, 72°C for 1 min). The pools were cleaned with 0.8 x volumetric ratio AMPure XP beads, were run with a High Sensitivity DNA5000 assay on a Tapestation 4200 (Agilent), and quantified using the Qubit high-sensitivity dsDNA assay. Seq-Well libraries were equimolarly pooled and clustered at 1.4pM concentration with 10% PhiX using High Output v2.1 chemistry on a NextSeq500 system. Sequencing was performed paired-end as followed: custom Drop-Seq Read 1 primer for 21 cycles, 8 cycles for the i7 index and 61 cycles for Read 2. Single-cell data were demultiplexed using bcl2fastq2 (v2.20).

### Processing of scRNA-seq raw data

For preprocessing, the generated fastq files from both Chromium™ and Seq-Well were loaded into a data pre-processing pipeline (version 0.31, available at https://github.com/Hoohm/dropSeqPipe) that relies on Drop-seq tools provided by the McCarroll lab (56). STAR alignment within the pipeline was performed using the human GENCODE reference genome and transcriptome hg38 release 27 (57). The resulting datasets were imported into R for further analyses.

For datasets for which TSO primers were used based on the Smart-Seq2 protocol, sequences starting with either the sequence 5’-GGG-3’, 5’-ATGGG-3’ or cell barcodes with a Hamming distance of 1 to 5’-ATGGG-3’ were excluded to avoid overlapping cell barcodes that are increased with this TSO primer. All other datasets were generated with the TSO primers as described in the original Seq-Well protocol. Next, datasets were examined for content of mitochondrial ribosomal transcripts. For further downstream analyses, the highly abundant mitochondrial transcripts *MT-RNR1* and *MT-RNR2* were excluded. The resulting datasets were then imported into the R package ‘Seurat’ [version 3.0.0 (58)] for downstream analyses.

### Quality control of scRNA-seq data

We defined cells and genes to be included for further analyses by the following criteria for each donor separately (1): Only genes that were found in at least 3 cells were kept (2); To retain granulocytes that contain only very limited number of transcripts, a relatively low threshold of 100 expressed genes was used to keep cells for further analyses (3); With regard to the rate of endogenous-to-mitochondrial counts per cell, blood cells with a rate > 5% and lavage cells with a rate >10% were excluded. For the comparison of scRNA-seq methods for clinical applications, these quality control filters resulted in a Chromium™ dataset of 13,909 cells (BALF = 7,960 cells; blood = 5,949 cells) across 22,701 genes and a Seq-Well dataset comprised of 34,622 cells (BALF = 20,106 cells; blood = 14,516 cells) across 21,644 genes. For the integrated analysis of Seq-Well data from COPD GOLD 2 patients and control donors, we obtained a Seq-Well dataset of 60,925 lavage cells across 25,348 genes and 54,569 blood cells across 23,056 genes (**Table S3**).

### Comparison of different single cell transcriptome technologies

We conducted a pilot experiment, in which we obtained single-cell RNA-sequencing (scRNA-seq) data using the most widely used droplet-based solution [Chromium from 10x Genomics (5)] and a well-based method [Seq-Well (6)]. After identification of cell-types based on marker gene expression of defined clusters (**Figure S1A**), we compared the cell populations between the two technologies. As ground truth, we characterized the cellular compartment in the alveolar space using multi-color flow cytometry (MCFC) (**Table S2**). All three approaches identified macrophages as the predominant cell type in the alveolar space (**Figure S1B**). When determining the cell type distribution for the droplet- and well-based scRNA-seq methods, granulocytes (neutrophils, eosinophils) were almost undetectable in the droplet-based method (**Figure S1B**).

### Dataset integration and dimensionality reduction of scRNA-seq data

If not stated otherwise, all following steps were conducted using the single-cell analysis pipeline Seurat. To account for variations in sequencing depth across cells, we applied a log-normalization strategy using CPM-normalization with a scale factor of 10,000. Next, the genes with the highest cell-to-cell variability in the dataset were determined by calculating the top 2,000 most variable genes by selecting the ‘vst’ method of the ‘FindVariableFeatures’ function in Seurat. For the comparison of scRNA-seq methods, the variable genes were determined separately for each technology, while for the integrated analysis of Seq-Well data from COPD GOLD 2 patients and control donors, variable genes were calculated separately for each donor.

To analyze the data without having any influence of batch effects resulting from either different donors or technologies, an integration approach based on ‘anchors’ across batches (59) was used to harmonize and integrate the different datasets by using the Seurat implementation with the default settings. After linear transformation of the remaining genes (scaling) to ensure homoscedasticity, the dimensionality of the data was reduced to 30 principal components (PCs) that was used as input for UMAP representation.

Next, doublet cells were identified utilizing the R package ‘DoubletFinder’ [version 2.0.2 (60)] by using the first 30 principal components of the non-integrated datasets, assuming a doublet formation rate of 10% and leaving all other parameters unaltered. The alleged duplicate cells were not removed from the dataset, but accumulations of these cells were highlighted and named accordingly. This procedure revealed, for example, that none of the identified macrophage states was defined by doublet cells (*data not shown*).

### Clustering of the integrated scRNA-seq datasets

The cellular heterogeneity of the integrated datasets was determined using a shared nearest neighbor (SNN)-graph based clustering algorithm implemented in the Seurat pipeline. For both the BALF and the blood data, we used the first 30 principle components as input and set the resolution to 0.7 and 0.6, respectively. The default setting for number of neighbors were used (k=20).

### Cell type annotation based on reference transcriptomic datasets

For cell annotation, we developed a slightly modified Python implementation of SingleR (61) (commit a4afed8, available at https://github.com/dviraran/SingleR) and an additional method called GenSigPro. We explicitly used two different methods with different reference datasets to capture variations in the annotation methods.

To compare and integrate these methods (with varying reference data), we first defined a common cell type standard to which all annotated cell types were matched. This standard, the mapping, and the actual reference data are available at FASTGenomics (https://beta.fastgenomics.org/p/bassler_scCOPD).

The SingleR method iteratively computes the bivariate correlations between the respective cluster expression vector and the multiple reference gene expression vectors for each cell type based on a set of differentially expressed (DE) genes. In each iteration, every cell type in the reference dataset is assigned a score based on these bivariate correlations with the different reference gene expression vectors of that cell type. The cell type with the lowest score is dropped and the DE genes among the remaining cell types are computed and, based on these genes, the bivariate correlations are computed again. This procedure thereby iteratively reduces the number of cell types until only one best fitting cell type is retained. We reimplemented the SingleR functionality to assign cell types per cluster in Python to use in our framework and in addition to the original algorithm, we included a threshold for the bivariate correlation score based on tests with randomized reference data. This made it possible to label cell clusters as “unknown” if the bivariate correlation score of the best fitting reference cell type was below 0.1 and thus no cell type could be assigned. As a reference for SingleR, we used data from both Blueprint+ENCODE (62, 63) and the Human Primary Cell Atlas (HPCA) (64). In addition to the implementation of the SingleR algorithm in Python, we also modified the reference datasets by reducing the reference to immune cells and lung tissue cells. Furthermore, based on the experimental setting of the reference dataset, we adapted some cell labels, e.g. the neutrophils were divided into mature, immature, and inflammatory neutrophils, whereas the original annotation had designated all these cells as neutrophils.

In order to capture potentially relevant variations in the annotation besides SingleR, we developed the similar but distinct statistical approach GenSigPro (Gene Signature Profiler) and incorporated a further reference dataset. To incorporate additional cell types, not included previously, we used manually curated reference data derived from the leukocyte expression dataset LM22 (65). This reference dataset encompasses one gene expression vector (signature) per cell type. While this made it incompatible to be used with SingleR, it allowed the reference dataset to be used in a multiple regression approach. The GenSigPro method fits a multiple linear regression for each cluster expression vector. The covariates in this regression are the reference expression vectors for each cell type that were obtained from the CIBERSORT algorithm (65). The more similar the cluster expression vector is to one of the reference expression vectors, the higher the regression coefficient for the respective reference vector. If the highest regression vector is positive and above an uncorrected significance threshold of α = 0.05, the cluster is assigned the respective cell-type label of this reference cell type, otherwise, the cluster is labeled “Unassigned”. To calculate the regression vectors, we used the Generalized Linear Model (GLM) with an added intercept from statsmodels [version 0.9.0 (66)] with the Gaussian family and left all parameters at their defaults.

GenSigPro does not alter the gene expression vectors during the annotation process, which is in contrast to SingleR where differentially expressed genes are calculated for each iteration. This is especially wanted for manually curated gene lists, like the used LM22 reference, where only high-confidence genes are included. Furthermore, whereas SingleR iteratively selects bivariate correlations, GenSigPro includes all reference gene vectors in a combined model. This allows us to assess the unique contributions of one reference gene vector over the others.

Using this different approach and manually curated reference data, also created more heterogeneous training data for the final consolidation by machine learning (see below). As reference data, we used a manually curated version of the leukocyte expression dataset LM22 (65), where the neutrophils were subdivided according to their activation state. We calculated the reference expression vectors by running CIBERSORT (version 1.06) on the modified LM22 dataset, leaving the default settings unchanged and setting the option “Filter non-hematopoietic genes from the signature matrix during construction”. The obtained signature genes (derived from the calculated support vectors) were almost completely (>99%; *data not shown*) contained in the signature genes of the original CIBERSORT publication (65). As this reference data only provides a mean expression vector per cell type, it was not suitable to be used with the SingleR approach.

Although both SingleR and GenSigPro can be applied also to vectors of single-cell expressions, we applied it to the mean of expression vectors within a cluster for more robust results. Since both GenSigPro and the modified SingleR are Python implementations, we performed clustering using the Louvain-clustering (67) function of Scanpy (68) by setting the number of neighbors to 24 and leaving the remaining parameters unaltered.

To assess the uncertainty of the annotation results, we added bootstrapping to GenSigPro and SingleR. The basic principle of bootstrapping is to create an artificial dataset by sampling subjects, in our case cells, with replacement such that in the resulting artificial dataset some cells will be excluded, whereas others will be included more than once. The analyses are then repeated on multiple of these artificial datasets, resulting in somewhat different results. For robust and certain patterns, different bootstrapped datasets generate similar results, while for random fluctuations different bootstraps result in highly different outcomes. Here, we conducted all cell typing analyses using 100 bootstrapped datasets.

### Cell type annotation using machine learning

To aggregate and consolidate the initial cell type annotation, we trained a Gradient Boosting Classifier on the combined data of all datasets to classify each cell into a cell type. Gradient Boosting is a machine learning technique that combines multiple classification trees in order to assign an input to different classes. This method is highly flexible and robust in the classification task and has high predictive power. We used an implementation of the Gradient Boosting algorithm from scikit-learn [version 0.19.1 (69)], the leading machine learning library for Python. For training the model, we used the raw gene expression matrix of each cell as input feature for the classification. We additionally extracted features from the data such as the type of tissue, the number of genes per cell, counts per cell, and the percentage of mitochondrial gene expression per cell. The training target of this model were the three cell type labels from GenSigPro and SingleR (Blueprint+Encode and HPCA). For this, we triplicated the data such that each cell with its feature vector was included three times, each with one label of the three cell-type annotations. Our aim was to apply the classifier to all cells in our data. However, as no distinct training data were available, we conducted a 3-fold cross-validation. In this procedure, two random thirds of a data set were used as training data, and the model assigned cell type names to the remaining cells. Importantly, a cell with all three cell type labels was only assigned either to the test or the training dataset. A major advantage of this machine learning method is that the classifier learns the specific expression profile of cell types and can take any cell type annotation as input, independent of techniques, such as bulk RNA-seq or microarray used as initial cell type annotation reference. In addition, we were able to apply the classifier at the single-cell level instead of the cluster mean expression level and thus achieved a higher resolution to exploit the full potential of scRNA-seq. This also allowed us to detect cell types with very low frequency in individual patients. Normally, these cells might end up in larger clusters with a different cell type and are therefore not detected. For all these reasons, this machine learning-based cell type annotation is unbiased, reliable, reproducible and scalable.

### Marker gene identification of scRNA-seq data

DE genes between identified cell types/clusters (referred to as marker genes) were defined using a Wilcoxon rank sum test for differential gene expression implemented in Seurat. The significance threshold for marker genes were set to an adjusted p-value smaller than 0.001 and the logarithmic fold change cutoff to at least 0.4. In addition, the detected marker genes should have been expressed in at least 50% of the cells within the respective cell types/clusters. Visualization of the obtained marker genes were mainly done using Seurat functions, such as dot plot representation of cell type-/cluster-specific marker gene expression or heatmap representation of marker genes across single cells. A more global overview of the expression profiles was obtained by calculating the mean expression values of marker genes per clusters, followed by scaling and centering of these values and representing them in a heatmap graph using the R package ‘pheatmap’ (version 1.0.12, https://CRAN.R-project.org/package=pheatmap), in which the genes were clustered according to the ‘ward.D’ agglomeration method.

Similar to the clustering and marker gene identification of the complete BALF dataset, we performed the same steps also for the detailed characterization of the macrophage population. In addition, we assessed the reproducibility of the identified clusters in the Seq-Well dataset. For this purpose, we used the BALF cells of the Chromium™ dataset. To consider possible influences of data integration on cell clustering, we used a different integration method, namely Harmony [version 1.0 (70)]. For determining the similarities between the Chromium™ and Seq-Well clusters, we calculated marker genes and assessed the overlap of the genes per cluster using the matchScore2 package [version 0.1.0 (71)]. For the majority of clusters, we found strong concordance between the Chromium™ and Seq-Well clusters (*data not shown*).

### Four-step cell type annotation

For the final cell type annotation of the integrated 61K BALF and the 55K blood dataset we used a four-step strategy for cell annotation and for the identification and finally removal of cells of inferior quality. The steps of the strategy include 1) the machine learning-based classifier, 2) cell clustering, followed by 3) a manual classifier-to-cluster comparison and 4) cluster-level marker gene analysis, including cleanup.

As the first step, the machine learning-based strategy is used to assign the most likely cell type to each cell in the dataset (**Figure S1E**). To determine the validity of this approach, we needed a dataset, for which the ground truth of the cell type is known by a secondary method, e g. flow cytometry data. In order to test the validity of machine learning-based strategy we generated a benchmark dataset. The data were obtained by fluorescence-activated cell sorting of blood-derived immune cells using cell type-specific markers followed by SMART-seq2 single-cell sequencing, which gives flow cytometry (ground truth) and scRNA-seq data information for each cell. In this validation experiment for the computational method only cells were used for which RNA expression values of typical cell markers were available (**Figure S1F**). Neither SingleR nor GenSigPro alone were able to correctly annotate all cells within the benchmark dataset due to incomplete cell type annotation within the reference (**Figure S1C**) used in these approaches. In contrast, the machine learning-based cell classifier was successful in consolidating the annotation results and thus resolving the different cell types in the blood scRNA-seq based benchmark dataset (**Figure S1F**). Applying the machine learning-based cell annotation to the integrated BALF dataset revealed all major immune cell types and for some cell types a subset structure (**Figure S1G**).

The second step consisted of clustering of the data in 18 main clusters (**Figure 1B**), which agreed with the areas that were enriched for distinct cell types predicted by the classifier (**Figure S1G**). However, we also found some cells that were annotated, *e.g.* as dendritic cells (DCs) (**Figure S1H**), which scattered away from the other DCs.

In the third step of the cell type annotation procedure, we determined which cell type occurred most frequently per main cluster according to the machine learning-based annotation and compared it with the identified marker genes for each cluster (**Figure 1D**) (step four). The application of this approach to all 18 major clusters in the BALF dataset led to a detailed resolution of the immune landscape in the alveolar space and this was similarly achieved for the blood dataset.

### “Gene set distance” analysis of annotated cell types (GO-shuffling)

This approach takes as input the average gene expression values per macrophage state of each patient and determines which functional gene sets, such as those based on gene ontology (GO) or pathway annotations, explain the strongest separation of COPD patients from controls in the Euclidean space. Gene set annotations were downloaded from the Molecular Signatures Database v7.0 (MSigDB) and comprised gene sets from the Kyoto Encyclopedia of Genes and Genomes (KEGG) (72) database, the Pathway Interaction Database (PID) (73), the Reactome Pathway database (74), Hallmark gene sets (75), BioCarta Pathways (76) and Gene Ontology (GO) (77, 78). In addition, we retrieved gene sets from WikiPathways (79). This search strategy resulted in a list of 12,755 gene sets, each containing a unique gene set term and a set of associated gene symbols.

As input, normalized scRNA-seq data was used, in which the cells were annotated according to the four-step cell-type annotation approach described above. Cell types containing at least 10 cells for each patient were retained and genes expressed in less than 5% of the cells in the respective cell type were excluded.

For each of the 12,755 gene sets, the “gene set distance” was calculated as follows for each cell type: Gene sets were taken into account that were present with a minimum of 3 genes. For each gene set, the Euclidean distance between all donors was calculated using the get_dist function from the R package ‘factoextra’ (version 1.0.5). Next, the mean distance of COPD patients, the mean distance of controls and the overall mean distance was calculated. The “gene set distance” was then defined as the overall mean distance divided by the mean distance of COPD patients plus the mean distance of control patients.


$$genesetdistance=\frac{dist_{overall}}{dist_{COPD}+dist_{CTRL}}$$

This metric allows to determine for which gene set the quotient takes a value close to or greater than 1, which means that the distance within the groups (COPD (*dist_COPD_*) or control (*dist_CTRL_*)) is smaller than the overall distance (*dist_overall_*) and consequently the distance is mainly defined by the difference between the groups. Since the Euclidean distance metric is prone to be affected by outliers in higher dimensions, we also tested this approach by using the Manhattan distance and got comparable results. For each cell type, we ranked the gene sets by their gene set distance. Visualization of the most frequent terms contained in the upper percentile of the predicted gene sets in the macrophage states was performed using the R package ‘wordcloud’ (version 2.6), in which filler and connective words were excluded. Alternatively, the gene sets in the upper percentile were filtered for association with ‘NOTCH’ or ‘lipidomics’ and the expression of the involved genes visualized in a heatmap.

### Modeling of metabolic pathways based on scRNA-seq data

The metabolic landscape of macrophage states was modeled using the Compass method [version 0.9.5 (10, 80)] by leaving the standard settings unaltered (model: RECON2 (81); lambda: 0; media: media1, which represents a rich extracellular medium, as defined in the Compass manuscript). As input, we simplified the single-cell data of the macrophages by using the ‘applyMicroClustering’ function of the R package ‘VISION’ [version 2.1.0 (82)], resulting in approximately 20 microclusters per patient. Next, we applied Compass to the microclusters for each donor separately. The output tables representing Compass scores for single reactions and synthesis of single metabolites of the individual donors were imported into R. They were concatenated and finally transformed as described in the Compass manuscript, except for disabling the division into meta-reactions. In detail, the concatenated output table *x* was first negatively log-transformed (*y* = -log(1+*x*)), the global minimum value of table *y* was subtracted from the values (*z* = *y* - min(*y*)) and the resulting table *z* was then used for further analysis. To determine which reactions and metabolites are significantly different between control donors and COPD patients, with the differences being reproducible in the COPD population, we performed Wilcoxon rank sum tests on Compass scores. We first computed the Wilcoxon p-value for every patient separately against all controls, took the median of these p-values, and kept reactions/metabolites for which -log10(median p-value) ≥ 2.5. We derived a second list of reactions and metabolites by similarly comparing control donors separately against all patients. The reactions and metabolites that have significant differences are the union of these two lists. Next, we excluded reactions with the lowest confidence score in the metabolic reconstruction (83), i.e., we discarded reactions with a confidence score of 1 and kept confidence scores of 2-4 (as well as 0 which is reserved for unannotated confidence). We also excluded metabolites that localize to cellular compartments other than the cytoplasm [c], extracellular space [e] or mitochondria [m]. Finally, the remaining reactions and metabolites were annotated using the Virtual Metabolic Human (VMH) database (84) and visualized in a heat map.

### Cell cycle state analysis of scRNA-Seq data

To categorize the cells within the macrophage states into the respective cell cycle states, we applied the ‘CellCycleScoring’ function of Seurat and substantiated the results using the ‘cyclone’ function (85) implemented in the R package ‘scran’ (version 1.10.2 (86)).

### Gene set variation analysis

To predict the functions of the macrophage states, we performed gene set variation analysis (GSVA) (87) by using the R package ‘GSVA’ (version 1.30.0) and defining ‘Poisson’ for the non-parametric estimation of the cumulative distribution function of expression levels across donors. For the GSVA input expression table, we calculated the sum of the expression of normalized scRNA-seq data for each patient in any macrophage state. As gene sets we used the gene set collection described in the section ‘GO-shuffling’ and additionally included the ‘ImmuneSigDB’ collection of MsigDB, whereby this collection was reduced to gene sets that had one of the following terms in the gene set description: ‘Mono’, ‘Macro’, ‘MDC’, ‘MDM’, ‘Dend’ and ‘DC’. This resulted in 14,160 gene sets. Similar to GO-shuffling, we filtered this collection for gene sets that were present with a minimum of 3 genes in a respective macrophage state. We applied an additional filter step to increase the stringency of the analysis. Therefore, we retained only gene sets in which the sum of the genes contained in the set were expressed in more than 30% of a macrophage state. The GSVA results per donor were combined for the respective macrophage state using a Borda rank and the top 250 ranked gene sets per subtype were visualized in an UpSet plot using the R package ‘UpSetR’ [version 1.3.3 (88)].

### AUCell for gene set enrichment analysis

Enrichment of gene sets was performed using the ‘AUCell’ method (89) implemented in the package (version 1.4.1) in R. We set the threshold for the calculation of the area under the curve (AUC) to the top 3% of the ranked genes and normalized the maximum possible AUC to 1. The resulting AUC values were subsequently visualized in a violin plot. For statistical testing, a Dunn’s *Post-Hoc* test using the “dunn.test” R package (version 1.3.5) was performed. Resulting p-values were corrected for multiple testing using the Benjamini-Hochberg method. This approach was used, for example, in **Figure 6A** to assess the enrichment of monocyte-derived macrophage signature genes provided by Wohnhaas (unpublished results). This signature was obtained from scRNA-seq data of monocyte-derived macrophages that were identified in BALF of a murine 12-week smoke model. Human orthologues (obtained from BioMart [version 2.42.0 (90)] of the murine marker genes were used for the enrichment analysis. In a similar way, we also performed the enrichment of monocyte-derived macrophage signatures obtained by Jaitin et al. (25) and Kim et al. (91).

### Distribution-free DE analysis across patient groups

To analyze the differences between the patient and control cohort, we employed a distribution-free test that preserves patient and cell information and thus considered possible individual donor effects. In contrast to available methods, it avoids the use of mini-bulk, the pooling of cells from different patients, and distribution assumptions. As input, we use the afore-computed macrophage state information and the normalized (non-integrated) scRNA-seq data.

For each macrophage state, a DE analysis between patient and control cohort was performed. Therefore, donors not possessing cells in a cluster – which happened in a few cases – and genes expressed in less than 10% of cells were disregarded for the analysis of this cluster. For each gene, the differences between all possible pairs of patients and controls were assessed using the non-parametric Wilcoxon rank sum test. To assess the differences between patient groups, the median Wilcoxon score of the pairwise tests was considered as a test statistic.

The Wilcoxon rank sum test was chosen because it does not rely on a specific distribution assumption. This is beneficial as the distribution of single-cell expressions is often skewed or shows multiple modes. Furthermore, benchmarking studies revealed that the Wilcoxon rank sum test performs well for the comparisons between two single cell data sets (92, 93).

To assess if the observed value of the test statistic was significant, the probability of observing an equal or more extreme value of the test statistic under the null hypothesis was evaluated. The null hypothesis was that there is no difference between the two groups. The null distribution was evaluated with the permutation test, taking all possible permutations into account. For all permutations the afore-described test statistic – the median Wilcoxon score – was evaluated. The distribution of the test statistic over all permutations provided the null distribution, since reshuffling of patients should not be significant under the null hypothesis. The p-value for the observed group assignment was then the fraction of permutations that led to an equal or more extreme value of the test statistic than the value of the test statistic of the observed patient arrangement.

### Testing/Simulation study of the DE method

The DE analysis method was evaluated using simulation data. A first evaluation – denoted as (I) – showed a good detection of differences in distributions across groups (patients and controls) with a similar mean. A second evaluation – denoted as (II) – indicated that there is no tendency to false positive discoveries if the distributions across groups are similar.

The simulation study was performed on the basis of the here examined COPD dataset. The number of individuals per group and sample sizes per individual were adopted from the original dataset. Sample sizes (number of cells per patient) were taken from the macrophage clusters 0, 1, 3 (**Table S3**).

The mean of the read count data per gene per individual is sampled from the same log-normal distribution, to ensure variability between the individuals,


$$\mu_{pati}^{group1},\mu_{patj}^{group2}∼logN\left(m,s\right),\text{with }m=1,\;s=0.15.$$

Single-cell read count data was then sampled from the negative binomial distribution, with the beforehand sampled means $\mu_{pati}^{group1},\mu_{patj}^{group2}$


$$Counts_{pati}^{group1}∼NB\left(h\left(\mu_{pati}^{group1},\sigma_{1}\right),\sigma_{1}\right)$$

$$Counts_{patj}^{group2}∼NB\left(h\left(\mu_{patj}^{group2},\sigma_{2}\right),\sigma_{2}\right),\text{ with }h\left(\mu ,\sigma \right)=\;\mu \cdot \sigma /\left(1-\sigma \right),$$

with *h* (*μ*, *σ*) being the number of successes, and *σ* the success probability.

For simulating differences in the distributions between the two groups (I), distinct success probability parameters (σ_1_, σ_2_) were used. Various combinations of σ_1_ and σ_2_ were considered to explore the properties of the methods. For simulating similar distributions (II) the success probability parameters were set to the same value (σ_1_=σ_2_). σ values were set within the interval [0.1, 0.9].

For each combination of σ_1_ and σ_2_, read count data was simulated for 50 μ’s per individual, which is in the following called a ‘set’. In total, for each σ-combination, three sets of read count data were simulated. DE analysis was performed with the proposed DE method and for a comparison with the widely used method edgeR [version 3.28.1 (94)], for each set of simulated read count data.

For the case of different distributions between the groups (I), the false negative rate (FNR) was calculated for each set of simulated read count data while evaluating the percentage of genes with p>0.05. In general, the proposed method identified the differences between the distributions for the two groups (low FNR), whereas edgeR failed to identify these differences for all cases with a FNR of over 90%. If the discrepancies between σ_1_ and σ_2_ were sufficiently large, which led to a clear difference between distributions (e.g. combinations 1-5 in **Table S5**), the proposed method performed well and achieved low FNRs. For similar values of σ_1_ and σ_2_ (e.g. combinations 6-8 in **Table S5**), the differences are – as expected – more difficult to identify and the FNR increases. The combination 9 was an exception, since for very small σ-values (here σ_1 =_ 0.086), sampling from the negative binomial distribution results in many zero counts, which then also leads to a clear characteristic of the distribution. Comparing the results between the distinct sample size combinations, the DE analysis on the simulated data sets with the sample size combinations 1 and 2 performed comparably well, whereas with sample size combination 3, which contained the lowest numbers of cells, performed slightly worse. For the case of similar distributions between the groups (II), the false positive rate (FPR) was evaluated with the percentage of genes with p<0.05, thus falsely detecting a difference, whereby no difference exists. For all implemented combinations both methods performed comparably well, whereas the proposed method showed slightly higher FPR, on average 1.47% higher (mean of edgeR: 3.77%, mean of proposed method: 5.24%).

To confirm the validity of this method, we performed a simulation study (see detailed methodological description below) in which we simulated gene expressions with similar mean expression values between COPD and control donors, but categorized gene expressions into two groups: 1) with different distributions between COPD and controls; or 2) with equal distributions between COPD and controls (**Figure S4B**). In comparison to the widely used DE method edgeR ^23^, the rate of false-positively identified DE genes was comparable to our DE method (**Figure S4C**), however, our method showed a significantly lower rate of false-negative results (**Figure S4D**). This means that our proposed DE method is able to detect differences in distributions across patient groups even if the differences in mean values are small and mostly the shape of the distributions changed.

### Application of the novel DE analysis approach and GSEA

DE analysis was performed for all macrophage states and the results are provided in **Table S5**. For the classification of genes being significantly DE, a test statistic cutoff of 0.75 was chosen. Additionally, for each macrophage state, the DE genes were sorted ascendingly according to their p-values and the 300 top ranked genes were chosen. The visualization of which DE genes are found and shared in which macrophage state was performed using the UpSetR package in R.

Gene set enrichment analysis (GSEA) was performed to identify shared common biological functions by groups of DE genes. The web-tool ‘g:Profiler’ (version e98_eg45_p14_ce5b097 (95)) was used to perform the functional profiling of the DE genes of interest (genes fulfilling the cutoff criteria for DE genes in >2 macrophage states). As multiple-testing correction method, g:Profiler’s in-house g:SCS algorithm was chosen, which corrects for multiple tests that are dependent on each other, which holds true for the hierarchically arranged GO terms. The analysis was done using the Gene Ontology (77, 78) database, as well as biological pathway databases, like KEGG (72), Reactome (74) and WikiPathways (79).

### Use of publicly available bulk data for validation of results

To investigate whether human leukocyte antigen (HLA) genes, which we found downregulated in macrophages in our single cell data, showed the same trend in a second cohort, we used a bulk transcriptome dataset of human macrophages (GSE13896 (17)), comprising samples from 39 non-smokers, 49 smokers, and 12 COPD patients. We filtered the normalized genes for HLA genes and visualized them as a box plot comparing non-smokers vs. smokers, non-smokers vs. COPD, and COPD vs. smokers using a Wilcoxon rank sum test, as provided in the R package stats. Additionally, to show whether the HLA genes shows statistically significant differences in enrichment between non-smokers and smokers, non-smokers and COPD, and COPD and smokers, we performed GSEA for the respective comparisons using the function GSEA with 10,000 permutations and Benjamini and Hochberg to control the false discovery rate from the package clusterProfiler (version 3.16.1 (96)). The normalized enrichment score (NES) was plotted on the x-axis where a negative NES shows an enrichment on the left hand side and a positive NES shows an enrichment on the right hand side of the plot. The significance of the enrichment was color coded using a negative log10 scale where values above 1.3 were considered as significant.

### Cell-Cell Communication

Potential cell-cell-interactions were inferred using ‘CellPhoneDB’ [version 2.1.1 (19, 97)]. As input, we used the normalized gene expression matrix of control and COPD patients that was filtered separately for cell types, which were defined by the four-step cell type annotation approach and identified in at least three patients of any group (COPD or control) and contained ≥ 10 cells per patient. Genes were filtered for being expressed in ≥ 5% of a respective cell type. To run CellPhoneDB, the following parameters were set: –iterations=1,000 –pvalue=0.1 –result-precision=10.

In order to visualize the cell-cell communication, we filtered for significant interactions (adjusted (Holm) p-value < 0.05) and summarized the interactions per cell type pair. Network visualizations were done with the ‘ggraph’ package (version 1.0.2) setting the layout to “fr”. To visualize single receptor-ligand pairs, we filtered for group-specific interactions (-log10(p-value) > 1) and visualized the resulting interactions for control and COPD.

To evaluate the downstream transcriptomic changes caused by cell-cell-interactions, we applied ‘NicheNet’ [version 0.1.0 (20, 98)]. As the CellPhoneDB analysis revealed a central role of the *C1Q* and monocyte-like macrophages in the cellular communication in BALF, we focused on these cells for the subsequent analysis. As the model in NicheNet is based on a different collection of databases than CellPhoneDB, we defined potential sender cell-receiver cell interactions independently of CellPhoneDB. As potential ligands, we accepted all genes that were expressed in >5% of any cell type within the COPD group and which matched at least one receptor from the genes expressed in > 5% of the *C1Q* macrophages or monocyte-like macrophages in the COPD group, respectively. As input genes to infer the ligand activity score from, we defined all DE genes with a median Wilcoxon score < (-0.75) and p-value of the median Wilcox score <0.05 for each state separately. As background genes, we defined all genes that are not DE in monocyte-like macrophages (or *C1Q* macrophages) and expressed in > 5% of monocyte-like macrophages (or *C1Q* macrophages). For ligand prioritization, we selected the top 3 genes with the highest AUPR from each of the comparisons resulting in 6 top ligands.

The expression of these ligands for each cell type was visualized in a heat map scaled by each gene. The target genes of all top ligands were visualized in a heat map with their regulatory potential score for each ligand and their mean expression in C1Q macrophages or monocyte-like macrophages for either COPD or control patients (scaled by gene). To further decipher the exact connection between the ligand and the target genes, we visualized the transcriptional network based on which NicheNet associated the target genes with *TGFB1* in a network with free topology. This network was subdivided into receptors for *TGFB1*, transcriptional regulators between *TGFB1* and the target genes. The connections were subdivided into signaling (which does not induce a direct transcriptional change) and transcriptional regulation.

### Monocyte-to-macrophage trajectory analysis

To generate a joint embedding of BAL and blood samples, the data were jointly pre-processed using ‘Scanpy’ [version 1.4.3 commit 0075c62 (68)] on AnnData (version 0.6.22.post2 commit 72c2bde). In concordance with previous analysis, cells from BALF were filtered out if the fraction of mitochondrial reads exceeded 0.1, and a threshold of 0.05 was used for blood samples. Genes that were expressed in fewer than 200 cells were also filtered out. Following previously published best-practices (99) we used scran normalization *via* the computeSumFactors function on the joint object. Spliced and unspliced counts were mapped to this object using scVelo [version 0.1.24 commit e45a65a (26)]. Quality control for spliced and unspliced counts was performed by removing cells with fewer than 20 spliced and/or 10 unspliced counts. Subsequent normalization by total counts and log-transformation was performed *via* the filter_and_normalize function from scVelo. Subsetting only relevant monocyte and macrophage populations from blood and BAL datasets (according to the coarse mapping shown in **Figure S6A**) resulted in a dataset of 57,280 cells and 11,530 genes.

The joint embedding of BAL and blood cells was generated by taking the top 4000 highly variable genes (HVGs) that were shared by most batches. This was done using the hvg_batch function from the single-cell data integration benchmarking package scIB [https://www.github.com/theislab/scib (100)]. This function computes the top 4000 HVGs per batch (here: donor) using Scanpy’s highly_variable_genes function with method cell_ranger. These genes are ranked by the number of batches in which each gene is highly variable, and by their mean index of dispersion across all batches. Using this ranked list, we selected the top 4000 genes as a representation of HVGs that are shared across batches. This constitutes a weak integration across batches without direct alteration of the transcriptome data.

Due to an observed batch effect when performing RNA velocity analysis across patients, we ran scVelo per patient and aggregated the individual patient velocities to create a joint velocity embedding. For each donor spliced and unspliced counts were smoothed using the moments function, velocity genes were selected by a stringent log likelihood threshold of 0.1 (between 45 and 172 genes per donor), and the dynamical scVelo model was fit. The resulting inferred single-cell velocities were projected onto the joint UMAP computed from all donors by running velocity_graph on the concatenated object.

Furthermore, partition-based graph abstraction [PAGA (27)] was used to assess the connectivity of cell identity clusters that were suggested to show transitions by RNA velocity. To robustly assess the connectivity of cell identity clusters across donors, we performed PAGA analysis per donor. We computed a kNN graph with Scanpy’s neighbors function (k=15) per donor using the joint PCA embedding across donors and ran the paga function on this graph. We used the resulting PAGA connectivities as a statistical test of kNN-graph connectivity between clusters. The median of PAGA connectivities over all donors with both blood and BAL samples was used as a PAGA distance metric.

## Data visualization

In general, Seurat and the ggplot2 package [version 3.1.0 (101)] was used to generate figures. For the monocyte-to-macrophage analysis Scanpy, UMAP and scVelo packages were used to generate figures. The graphical summary was created with BioRender.com.

## Quantification and statistical analysis

If not otherwise stated, the statistical evaluation was carried out in relation to the total sample size *n*. A t-test (two-sided) was used for *n* ≤ 10, otherwise a Wilcoxon rank-sum test was used.

## Code availability

We deposited the code for the novel DE analysis approach used in this study on Zenodo (https://doi.org/10.5281/zenodo.3717776). The analysis code used to generate the majority of the figures are available *via* FASTGenomics (https://beta.fastgenomics.org/p/bassler_scCOPD).

## Data availability statement

The original contributions presented in the study are publicly available. This data can be found here: https://ega-archive.org/studies/EGAS00001004369.

## Ethics statement

The studies involving human participants were reviewed and approved by Ethics committees of the University of Bonn and University hospital Bonn. The patients/participants provided their written informed consent to participate in this study.

## Author contributions

Conceptualization, KB DS, PB, and JS. Methodology, KB, SW-H, JS, BR, FB, HD, JH, ML, and FT. Software: KB, SW-H, BR, ED, and ML. Investigation, KB, WF, TK, AH, BR, ED, ML, NR, CO-S, SW-H, LB, PG, MB, KH, HT, MK, HF, JS-S, EH, CT, AF, DT, AA, and TU. Biospecimen/enzyme resources, CP, TS, DS, IK and MG. Writing – Original Draft, KB and JS. Writing – Review & Editing, KB, JS, WF, TK, AH, BR, ED, ML, NR, CO-S, SW-H, AW, LB, PG, MB, CW, MW, TH, JS-S, EH, IK, MG, CT, AS, HD, MB, PB, NY, AA, TU, JH, FT, DS. Visualization, KB, AH, NR, AA, and ML. Supervision, JS. Project Administration, JS. Funding Acquisition, WF, JS, FT, JH, NY, AS, MG, and MB. All authors contributed to the article and approved the submitted version.

## Funding

This work was supported in part by Boehringer Ingelheim, by the German Research Foundation (DFG) to J.L.S. (GRK 2168 (project number 272482170), INST 217/577-1, EXC2151/1 (ImmunoSensation2 - the immune sensory system, project number 390873048), project numbers 329123747, 347286815), by the HGF grant sparse2big to J.L.S. and F.J.T., the FASTGenomics grant of the German Federal Ministry for Economic Affairs and Energy to J.L.S., the EU projects SYSCID (grant number 733100), ERA CVD (grant number 00160389), and DiscovAIR (grant number 874656). W.F. was supported by a fellowship of the Alexander von Humboldt Foundation (JPN-1186019-HFST-P). J.H. and E.D. were supported by the Horizon2020 grant CanPathPro (grant number 686282). F.J.T. acknowledges support by the BMBF (grant# 01IS18036A and grant# 01IS18053A), by the Helmholtz Association (Incubator grant sparse2big, grant # ZT-I-0007) and by the Chan Zuckerberg Initiative DAF (advised fund of Silicon Valley Community Foundation, 182835). J.H. was supported by the Horizon2020 grant CanPathPro (grant number 686282). N.Y. and A.W. were supported by the Chan Zuckerberg Biohub and by a National Institute of Mental Health (NIMH) grant NIH5U19MH114821. A.K.S was supported by the Searle Scholars Program, the Beckman Young Investigator Program, the Pew-Stewart Scholars Program for Cancer Research, a Sloan Fellowship in Chemistry, the NIH (5U24AI118672, 1U54CA217377) and the Bill and Melinda Gates Foundation. This work is supported by grants from the DFG to M.G. (GE 976/9-2) and M.B. (Immunsensation2; EXC2151 – 390873048).

## Acknowledgments

We thank F. Gondorf for technical assistance with Seahorse, T. Quast and K. Zölzer for their support in microscopy procedures, and S. Mukherjee and B. Taschler for their support in the development of GO-shuffling.

## Conflict of interest

The handling editor [AH] declared a shared affiliation with the author(s) [AW, NY] at the time of review. BR, FB, MK and HD were employed by CommaSoft. CW and PB were employed by Boehringer Ingelheim.

The remaining authors declare that the research was conducted in the absence of any commercial or financial relationships that could be constructed as a potential conflict of interest.

## Publisher’s note

All claims expressed in this article are solely those of the authors and do not necessarily represent those of their affiliated organizations, or those of the publisher, the editors and the reviewers. Any product that may be evaluated in this article, or claim that may be made by its manufacturer, is not guaranteed or endorsed by the publisher.

## Supplementary material

Supplementary Figure 1 Characterization of BALF immune cells using appropriate scRNA-seq technology and four-step cell-type annotation strategy (related to ). **(A)** UMAP representation of integrated blood and BALF data from different patients and two scRNA-seq technologies (10x Chromium and Seq-Well). The UMAP is split by technology and colored according to identified clusters. Clusters were additionally assigned to cell-types based on marker gene expression (according to **Table S6**). **(B)** Stacked bar plots of the relative cell-type proportions for MCFC, which served as ground truth, and cell-type proportions based on the assigned clusters of the two scRNA-seq technologies. **(C)** Overview of the cell types contained in the reference files used for cell-type annotation. The orange color indicates that the respective cell type is included in the reference file. **(D)** Confusion plots showing the concordance between the respective cell-type annotations across different annotation methods (SingleR with the Blueprint + ENCODE or HPCA as reference and GenSigPro with signatures from the LM22 dataset as reference). Only cell types that can be found in all reference files as shown in **Figure S1C** are displayed. **(E)** Scheme of the gradient boosted decision tree-based machine learning-approach for cell-type annotation. **(F)** UMAP representation of a benchmarking blood immune cell dataset (according to **Table S1**). The cells in the UMAPs are colored according to the respective cell annotation methods. The ground truth is derived based on the unique cell-type marker gene expression of each cell. Accumulation of cells that are annotated by the respective annotation methods, but show a deviation in the annotation with respect to the ground truth, are marked with an arrow. **(G)** UMAP representation of integrated BALF data obtained from COPD patients and control donors *via* the Seq-Well technology. Coloring according to machine learning-based cell-type annotation. **(H)** UMAP representation of the integrated dataset with the coloring of DCs, as predicted by the machine learning-based cell-type annotation. Non-DCs are colored light gray. MCFC = multicolor flow cytometry; BALF = bronchoalveolar lavage fluid; DC = dendritic cell; MDM = monocyte-derived macrophage; SR1 = SingleR (HPCA); SR2 = SingleR (Blueprint + ENCODE); macro = macrophage; mono = monocyte.

Supplementary Figure 2 Characterization of identified macrophage states (related to ). **(A)** Bar plot representation of the proportion of cells in the respective cell cycle states per cluster (according to ). **(B)** Stacked bar plot showing the proportion of individual donors in each macrophage state. **(C)** Schematic workflow to predict the cellular functions of each cluster based on gene set variation analysis (GSVA). **(D)** UpSet plot of the GSVA results (according to **Figure S2C**). Terms of cellular functions found in the same clusters are grouped into bins and the size of the bins is represented as a bar plot on the right, with bins containing more than 25 terms (dashed line) colored red. On the left side, dots indicate which clusters contain and share the binned terms. Frequently occurring terms of cellular functions within the bins containing more than 25 terms are shown. **(E)** Violin plots (with marked median enrichment values) displaying enrichment of different gene sets across clusters based on the Area Under the Curve (AUC). BALF = bronchoalveolar lavage fluid; sign. = signature; MФ = macrophage; GSVA = gene set variation analysis; mito. = mitochondrial; degrad. = degradation; mod. = modification; present. = presentation.

Supplementary Figure 3 Characterization of altered lipid metabolism in macrophages of COPD patients (related to ). **(A)** Schematic workflow of the GO-shuffling approach. **(B)** Heat map of lipid metabolism-associated genes predicted by the GO-shuffling approach. The mean gene expression per donor is represented as a z-transformed value (across all donors). Columns and rows of the heat map are sorted by hierarchical clustering. Genes that have been described as causing cholesteryl accumulation through the dysfunctionality of their protein products are marked in yellow. **(C)** Schema of the key steps in cholesterol metabolism and storage. Metabolites predicted by Compass are highlighted with a gray background. Enzymes involved in metabolism are abbreviated with a number that identifies them also in **Figure S3B**. **(D)** Heat map of NOTCH-signaling associated genes predicted by the GO-shuffling approach. The mean gene expression per donor is represented as a z-transformed value (across all donors). Columns and rows of the heat map are sorted by hierarchical clustering. **(E)** Schema of the time-dependent course of the oxygen consumption rate (OCR) and the inferred mitochondrial parameters based on the injection of different compounds (shown at the top of the plot). dist = distance; visual = visualization.

Supplementary Figure 4 Benchmarking of the novel DE-analysis approach (related to ). **(A)** Schematic workflow of the permutation test-based DE analysis approach. **(B)** Workflow for the simulation study used for the evaluation of the performance for the proposed DE-method. Two cases are considered, single cell count data was simulated for multiple patients within two groups, with I) different distributions between the groups, II) similar distributions between the groups. For each patient, the mean of the read counts is sampled from the same log-normal distribution with m = 1 and s = 0.15. The read counts are sampled from a negative binomial distribution with success probability (σ) parameters dependent on the considered case. When simulating different distributions between the groups (I), distinct σ-values are assigned to each group. When simulating the same distributions for the groups (II), the same σ-values are assigned to the groups. The number of cells per patient were chosen according to the Seq-Well dataset, where three sample size combinations were considered, originating from three macrophage state datasets. **(C)** False positive rate (FPR) in simulation study (II) (percentage of genes with a p-value < 0.05) for the proposed method and edgeR for 5 σ combinations and for each sample size realization. Boxplots (with marked median values) comprise results of three repeated sets of simulated data (one set of simulated data: 50* μ per patient, per σ combination). Low FPR denotes a higher number of correct equally expressed gene classifications. **(D)** False negative rate (FNR) in simulation study (I) (percentage of genes with a p-value > 0.05) for the proposed method and edgeR for 9 σ-combinations and for each sample size realization. Boxplots (with marked median values) comprise results of three repeated sets of simulated data (one set of simulated data: 50* μ per patient, per σ combination). Low FNR denotes a higher number of correct differential expressed gene classifications. **(E)** Violin plot with marked median of the HLA-DRA/-DRB1/-DPA1/-DPB1/-DQB1 and -DRB5 expression in macrophages based on scRNA-seq data. The plot shows the expression across the donors, whereby the donors were downsampled to the same number of cells, followed by downsampling to the same number of cells between COPD and control. The plot displays cells with an expression > 0. **(F)** Fluorescence intensity histograms showing representative samples of flow cytometric analysis of HLA-DR expression on the cell surface of isolated macrophages (FMO = fluorescence minus one). **(G)** Box plots with marked median of the calculated effect sizes of HLA-DR expression in COPD and control with the representation of individual donors (control n = 8, COPD n = 5; error bars indicating the standard deviation; statistics based on the Wilcoxon rank sum test). FPR = false positive rate; FNR = false negative rate; DE = differential expression; μ = sampled mean; logN(m,s) = log-normal distribution with mean m and standard deviation s; Count = sampled single cell read count data; NB(h,σ) = negative binomial distribution with: h = number of successes, σ = success probability parameter; proliferat. = proliferating; FMO = fluorescence minus one.

Supplementary Figure 5 Investigation of cell-to-cell interactions to infer important signaling pathways in macrophages (related to ). **(A)** Dot plot representation of monocyte-like macrophage-dependent ligand-receptor interactions predicted by CellPhoneDB that show significant enrichment (represented by the p-value) of the interacting pair in the interacting cell types either in COPD or in the control. Depicted are only selected interactions. **(B, C)** Illustration of the selection of potential upstream ligands of monocyte-like macrophages or C1Q+ macrophages based on the NicheNet analysis. The histograms show distributions based on ligand activity derived from the area under the precision recall curve (AUPR, upper histogram) and the Pearson correlation coefficient (PCC, lower histogram). The ligand activity of the highest ranked ligands is displayed in a color code together with the names of the 20 highest ranked ligands. The ligands predicted by the CellPhoneDB analysis (according to **Figure S5A**) are highlighted in red and the top 3 ligands based on AUPR for either monocyte-like macrophages or C1Q+ macrophages (as presented in ) are underlined. **(D)** Expression of ligand targets from in macrophage subsets comparing COPD and control patients (z-transformed by gene). AUPR = area under the precision recall curve; PCC = Pearson correlation coefficient; MФ = macrophage; DC = dendritic cell.

Supplementary Figure 6 Modeling the association of blood monocytes and BALF macrophages (related to ). **(A)** UMAP of embedded macrophages/monocytes from BALF and blood monocytes with coloring according to the cell types derived from the combined labels. The dendrogram on the right side illustrates the transcriptional relationship between the macrophage subtypes and shows how several subtypes were summarized in the combined labels. **(B)** Projection of computed RNA velocity vectors onto the UMAP of the embedded data. BALF = bronchoalveolar lavage fluid; mono = monocyte; MФ = macrophage; proliferat. = proliferating.

Supplementary Figure 7 Instructions for accessing the COPD Seq-Well dataset and scripts *via* the FASTGenomics platform.

## References

[1] GBD 2017 Causes of Death Collaborators. Global, regional, and national age-sex-specific mortality for 282 causes of death in 195 countries and territories, 1980-2017: A systematic analysis for the global burden of disease study 2017. Lancet (2018) 392:1736–88. doi: 10.1016/S0140-6736(18)32203-7 PMC6227606 30496103

[2] Celli BR Wedzicha JA. Update on clinical aspects of chronic obstructive pulmonary disease. N Engl J Med (2019) 381:1257–66. doi: 10.1056/NEJMra1900500 31553837

[3] Barnes PJ Burney PGJ Silverman EK Celli BR Vestbo J Wedzicha JA. Chronic obstructive pulmonary disease. Nat Rev Dis Primers (2015) 1:15076. doi: 10.1038/nrdp.2015.76 27189863

[4] Barnes PJ. Alveolar macrophages as orchestrators of COPD. COPD (2004) 1:59–70. doi: 10.1081/COPD-120028701 16997739

[5] Zheng GXY Terry JM Belgrader P Ryvkin P Bent ZW Wilson R. Massively parallel digital transcriptional profiling of single cells. Nat Commun (2017) 8:14049. doi: 10.1038/ncomms14049 28091601 PMC5241818

[6] Gierahn TM Wadsworth MH Hughes TK Bryson BD Butler A Satija R. Seq-well: Portable, low-cost RNA sequencing of single cells at high throughput. Nat Methods (2017) 14:395–8. doi: 10.1038/nmeth.4179 PMC5376227 28192419

[7] Wauters E Van Mol P Garg AD Jansen S Van Herck Y Vanderbeke L. Discriminating mild from critical COVID-19 by innate and adaptive immune single-cell profiling of bronchoalveolar lavages. Cell Res (2021) 0:1–19. doi: 10.1038/s41422-020-00455-9 PMC8027624 33473155

[8] Chan SM Weng AP Tibshirani R Aster JC Utz PJ. Notch signals positively regulate activity of the mTOR pathway in T-cell acute lymphoblastic leukemia. Blood (2007) 110:278–86. doi: 10.1182/blood-2006-08-039883 PMC1896117 17363738

[9] Alpert A Pickman Y Leipold M Rosenberg-Hasson Y Ji X Gaujoux R. A clinically meaningful metric of immune age derived from high-dimensional longitudinal monitoring. Nat Med (2019) 25:487–95. doi: 10.1038/s41591-019-0381-y PMC6686855 30842675

[10] Wagner A Wang C Fessler J DeTomaso D Avila-Pacheco J Kaminski J. Metabolic modeling of single Th17 cells reveals regulators of autoimmunity. Cell (2021) 184:4168–4185.e21. doi: 10.1016/j.cell.2021.05.045 34216539 PMC8621950

[11] Trapnell BC Nakata K Bonella F Campo I Griese M Hamilton J. Pulmonary alveolar proteinosis. Nat Rev Dis Primers (2019) 5:16. doi: 10.1038/s41572-019-0066-3 30846703

[12] de Aguiar Vallim TQ Lee E Merriott DJ Goulbourne CN Cheng J Cheng A. ABCG1 regulates pulmonary surfactant metabolism in mice and men. J Lipid Res (2017) 58:941–54. doi: 10.1194/jlr.M075101 PMC5408613 28264879

[13] Nugent AA Lin K van Lengerich B Lianoglou S Przybyla L Davis SS. TREM2 regulates microglial cholesterol metabolism upon chronic phagocytic challenge. Neuron (2020) 105:837–854.e9. doi: 10.1016/j.neuron.2019.12.007 31902528

[14] Deczkowska A Weiner A Amit I. The physiology, pathology, and potential therapeutic applications of the TREM2 signaling pathway. Cell (2020) 181:1207–17. doi: 10.1016/j.cell.2020.05.003 32531244

[15] O’Beirne SL Kikkers SA Oromendia C Salit J Rostmai MR Ballman KV. Alveolar macrophage immunometabolism and lung function impairment in smoking and chronic obstructive pulmonary disease. Am J Respir Crit Care Med (2020) 201:735–9. doi: 10.1164/rccm.201908-1683LE PMC7068819 31751151

[16] Boukhenouna S Wilson MA Bahmed K Kosmider B. Reactive oxygen species in chronic obstructive pulmonary disease. Oxid Med Cell Longev (2018) 2018:5730395. doi: 10.1155/2018/5730395 29599897 PMC5828402

[17] Shaykhiev R Krause A Salit J Strulovici-Barel Y Harvey B-G O’Connor TP. Smoking-dependent reprogramming of alveolar macrophage polarization: implication for pathogenesis of chronic obstructive pulmonary disease. J Immunol (2009) 183:2867–83. doi: 10.4049/jimmunol.0900473 PMC2873685 19635926

[18] Kaku Y Imaoka H Morimatsu Y Komohara Y Ohnishi K Oda H. Overexpression of CD163, CD204 and CD206 on alveolar macrophages in the lungs of patients with severe chronic obstructive pulmonary disease. PloS One (2014) 9:e87400. doi: 10.1371/journal.pone.0087400 24498098 PMC3907529

[19] Efremova M Vento-Tormo M Teichmann SA Vento-Tormo R. CellPhoneDB: inferring cell-cell communication from combined expression of multi-subunit ligand-receptor complexes. Nat Protoc (2020) 15:1484–506. doi: 10.1038/s41596-020-0292-x 32103204

[20] Browaeys R Saelens W Saeys Y. NicheNet: modeling intercellular communication by linking ligands to target genes. Nat Methods (2020) 17:159–62. doi: 10.1038/s41592-019-0667-5 31819264

[21] Verhamme FM Bracke KR Amatngalim GD Verleden GM Van Pottelberge GR Hiemstra PS. Role of activin-a in cigarette smoke-induced inflammation and COPD. Eur Respir J (2014) 43:1028–41. doi: 10.1183/09031936.00082413 24232707

[22] Takizawa H Tanaka M Takami K Ohtoshi T Ito K Satoh M. Increased expression of transforming growth factor-beta1 in small airway epithelium from tobacco smokers and patients with chronic obstructive pulmonary disease (COPD). Am J Respir Crit Care Med (2001) 163:1476–83. doi: 10.1164/ajrccm.163.6.9908135 11371421

[23] Yu X Buttgereit A Lelios I Utz SG Cansever D Becher B. The cytokine TGF-β promotes the development and homeostasis of alveolar macrophages. Immunity (2017) 47:903–912.e4. doi: 10.1016/j.immuni.2017.10.007 29126797

[24] Guilliams M Scott CL. Does niche competition determine the origin of tissue-resident macrophages? Nat Rev Immunol (2017) 17:451–60. doi: 10.1038/nri.2017.42 28461703

[25] Jaitin DA Adlung L Thaiss CA Weiner A Li B Descamps H. Lipid-associated macrophages control metabolic homeostasis in a Trem2-dependent manner. Cell (2019) 178:686–698.e14. doi: 10.1016/j.cell.2019.05.054 31257031 PMC7068689

[26] Bergen V Lange M Peidli S Wolf FA Theis FJ. Generalizing RNA velocity to transient cell states through dynamical modeling. Nat Biotechnol (2020) 38:1408–14. doi: 10.1038/s41587-020-0591-3 32747759

[27] Wolf FA Hamey FK Plass M Solana J Dahlin JS Göttgens B. PAGA: graph abstraction reconciles clustering with trajectory inference through a topology preserving map of single cells. Genome Biol (2019) 20:59. doi: 10.1186/s13059-019-1663-x 30890159 PMC6425583

[28] Sun Y Zhou J. New insights into early intervention of chronic obstructive pulmonary disease with mild airflow limitation. Int J Chron Obstruct Pulmon Dis (2019) 14:1119–25. doi: 10.2147/COPD.S205382 PMC6536809 31213792

[29] Kammerl IE Dann A Mossina A Brech D Lukas C Vosyka O. Impairment of immunoproteasome function by cigarette smoke and in chronic obstructive pulmonary disease. Am J Respir Crit Care Med (2016) 193:1230–41. doi: 10.1164/rccm.201506-1122OC 26756824

[30] Houssaini A Breau M Kebe K Abid S Marcos E Lipskaia L. mTOR pathway activation drives lung cell senescence and emphysema. JCI Insight (2018) 3(3):e93203. doi: 10.1172/jci.insight.93203 PMC5821218 29415880

[31] Barnes PJ. Senescence in COPD and its comorbidities. Annu Rev Physiol (2017) 79:517–39. doi: 10.1146/annurev-physiol-022516-034314 27959617

[32] Ryter SW Rosas IO Owen CA Martinez FJ Choi ME Lee CG. Mitochondrial dysfunction as a pathogenic mediator of chronic obstructive pulmonary disease and idiopathic pulmonary fibrosis. Ann Am Thorac Soc (2018) 15:S266–72. doi: 10.1513/AnnalsATS.201808-585MG PMC6944396 30759019

[33] Shaw AC Goldstein DR Montgomery RR. Age-dependent dysregulation of innate immunity. Nat Rev Immunol (2013) 13:875–87. doi: 10.1038/nri3547 PMC4096436 24157572

[34] Taylor AE Finney-Hayward TK Quint JK Thomas CMR Tudhope SJ Wedzicha JA. Defective macrophage phagocytosis of bacteria in COPD. Eur Respir J (2010) 35:1039–47. doi: 10.1183/09031936.00036709 19897561

[35] Paul P van den Hoorn T Jongsma MLM Bakker MJ Hengeveld R Janssen L. A genome-wide multidimensional RNAi screen reveals pathways controlling MHC class II antigen presentation. Cell (2011) 145:268–83. doi: 10.1016/j.cell.2011.03.023 21458045

[36] Ramdas V McBride M Denby L Baker AH. Canonical transforming growth factor-β signaling regulates disintegrin metalloprotease expression in experimental renal fibrosis *via* miR-29. Am J Pathol (2013) 183:1885–96. doi: 10.1016/j.ajpath.2013.08.027 PMC4188136 24103556

[37] Lagares D Ghassemi-Kakroodi P Tremblay C Santos A Probst CK Franklin A. ADAM10-mediated ephrin-B2 shedding promotes myofibroblast activation and organ fibrosis. Nat Med (2017) 23:1405–15. doi: 10.1038/nm.4419 PMC5720906 29058717

[38] Hashimoto D Chow A Noizat C Teo P Beasley MB Leboeuf M. Tissue-resident macrophages self-maintain locally throughout adult life with minimal contribution from circulating monocytes. Immunity (2013) 38:792–804. doi: 10.1016/j.immuni.2013.04.004 23601688 PMC3853406

[39] Schyns J Bai Q Ruscitti C Radermecker C De Schepper S Chakarov S. Non-classical tissue monocytes and two functionally distinct populations of interstitial macrophages populate the mouse lung. Nat Commun (2019) 10:3964. doi: 10.1038/s41467-019-11843-0 31481690 PMC6722135

[40] Fabbri LM Rabe KF. From COPD to chronic systemic inflammatory syndrome? Lancet (2007) 370:797–9. doi: 10.1016/S0140-6736(07)61383-X 17765529

[41] Mak JCW Chan-Yeung MMW Ho SP Chan KS Choo K Yee KS. Elevated plasma TGF-beta1 levels in patients with chronic obstructive pulmonary disease. Respir Med (2009) 103:1083–9. doi: 10.1016/j.rmed.2009.01.005 19186046

[42] Agustí A Hogg JC. Update on the pathogenesis of chronic obstructive pulmonary disease. N Engl J Med (2019) 381:1248–56. doi: 10.1056/NEJMra1900475 31553836

[43] Cottin V Nunes H Brillet PY Delaval P Devouassoux G Tillie-Leblond I. Combined pulmonary fibrosis and emphysema: A distinct underrecognised entity. Eur Respir J (2005) 26:586–93. doi: 10.1183/09031936.05.00021005 16204587

[44] Meyer KC Raghu G Baughman RP Brown KK Costabel U du Bois RM. An official American thoracic society clinical practice guideline: the clinical utility of bronchoalveolar lavage cellular analysis in interstitial lung disease. Am J Respir Crit Care Med (2012) 185:1004–14. doi: 10.1164/rccm.201202-0320ST 22550210

[45] Ellis PH B. flowCore. Bioconductor (2017). doi: 10.18129/b9.bioc.flowcore

[46] McInnes L Healy J. UMAP: Uniform manifold approximation and projection for dimension reduction. Journal of Open Source Software (2018) 3(29), 861. doi: 10.21105/joss.00861

[47] Becht E McInnes L Healy J Dutertre C-A Kwok IWH Ng LG. Dimensionality reduction for visualizing single-cell data using UMAP. Nat Biotechnol (2018) 37:38–44. doi: 10.1038/nbt.4314 30531897

[48] Parks DR Roederer M Moore WA. A new “Logicle” display method avoids deceptive effects of logarithmic scaling for low signals and compensated data. Cytometr A (2006) 69:541–51. doi: 10.1002/cyto.a.20258 16604519

[49] Levine JH Simonds EF Bendall SC Davis KL Amir ED Tadmor MD. Data-driven phenotypic dissection of AML reveals progenitor-like cells that correlate with prognosis. Cell (2015) 162:184–97. doi: 10.1016/j.cell.2015.05.047 PMC4508757 26095251

[50] Biosciences BD. Standardizing application setup across multiple flow cytometers using BD FACSDivaTM version 6 software. BD Biosciences Technical Bulletin New Jersey, USA (2012) p. 1–16.

[51] Njoroge JM Mitchell LB Centola M Kastner D Raffeld M Miller JL. Characterization of viable autofluorescent macrophages among cultured peripheral blood mononuclear cells. Cytometry (2001) 44(1):38–44. doi: 10.1002/1097-0320(20010501)44:1<38::AID-CYTO1080>3.0.CO;2-T 11309807

[52] Schneider CA Rasband WS Eliceiri KW. NIH Image to ImageJ: 25 years of image analysis. Nat Methods (2012) 9:671–5. doi: 10.1038/nmeth.2089 PMC5554542 22930834

[53] Herzog R Schuhmann K Schwudke D Sampaio JL Bornstein SR Schroeder M. LipidXplorer: a software for consensual cross-platform lipidomics. PloS One (2012) 7:e29851. doi: 10.1371/journal.pone.0029851 22272252 PMC3260173

[54] Ritchie ME Phipson B Wu D Hu Y Law CW Shi W. Limma powers differential expression analyses for RNA-sequencing and microarray studies. Nucleic Acids Res (2015) 43:e47. doi: 10.1093/nar/gkv007 25605792 PMC4402510

[55] Picelli S Björklund AK Reinius B Sagasser S Winberg G Sandberg R. Tn5 transposase and tagmentation procedures for massively scaled sequencing projects. Genome Res (2014) 24:2033–40. doi: 10.1101/gr.177881.114 PMC4248319 25079858

[56] Macosko EZ Basu A Satija R Nemesh J Shekhar K Goldman M. Highly parallel genome-wide expression profiling of individual cells using nanoliter droplets. Cell (2015) 161:1202–14. doi: 10.1016/j.cell.2015.05.002 PMC4481139 26000488

[57] Harrow J Frankish A Gonzalez JM Tapanari E Diekhans M Kokocinski F. GENCODE: The reference human genome annotation for the ENCODE project. Genome Res (2012) 22:1760–74. doi: 10.1101/gr.135350.111 PMC3431492 22955987

[58] Butler A Hoffman P Smibert P Papalexi E Satija R. Integrating single-cell transcriptomic data across different conditions, technologies, and species. Nat Biotechnol (2018) 36:411–20. doi: 10.1038/nbt.4096 PMC6700744 29608179

[59] Stuart T Butler A Hoffman P Hafemeister C Papalexi E Mauck WM. Comprehensive integration of single-cell data. Cell (2019) 177:1888–1902.e21. doi: 10.1016/j.cell.2019.05.031 31178118 PMC6687398

[60] McGinnis CS Murrow LM Gartner ZJ. DoubletFinder: Doublet detection in single-cell RNA sequencing data using artificial nearest neighbors. Cell Syst (2019) 8:329–337.e4. doi: 10.1016/j.cels.2019.03.003 30954475 PMC6853612

[61] Aran D Looney AP Liu L Wu E Fong V Hsu A. Reference-based analysis of lung single-cell sequencing reveals a transitional profibrotic macrophage. Nat Immunol (2019) 20:163–72. doi: 10.1038/s41590-018-0276-y PMC6340744 30643263

[62] Stunnenberg HG International Human Epigenome Consortium Hirst M. The international human epigenome consortium: A blueprint for scientific collaboration and discovery. Cell (2016) 167:1145–9. doi: 10.1016/j.cell.2016.11.007 27863232

[63] ENCODE Project Consortium. An integrated encyclopedia of DNA elements in the human genome. Nature (2012) 489:57–74. doi: 10.1038/nature11247 22955616 PMC3439153

[64] Mabbott NA Baillie JK Brown H Freeman TC Hume DA. An expression atlas of human primary cells: inference of gene function from coexpression networks. BMC Genomics (2013) 14:632. doi: 10.1186/1471-2164-14-632 24053356 PMC3849585

[65] Newman AM Liu CL Green MR Gentles AJ Feng W Xu Y. Robust enumeration of cell subsets from tissue expression profiles. Nat Methods (2015) 12:453–7. doi: 10.1038/nmeth.3337 PMC4739640 25822800

[66] Seabold S Perktold J. Statsmodels: Econometric and statistical modeling with Python. In: Proceedings of the 9th Python in science conference proceedings of the python in science conference. SciPy. (2010) Texas, USA. p. 92–6. doi: 10.25080/Majora-92bf1922-011

[67] Blondel VD Guillaume J-L Lambiotte R Lefebvre E. Fast unfolding of communities in large networks. J Stat Mech (2008) 2008:P10008. doi: 10.1088/1742-5468/2008/10/P10008

[68] Wolf FA Angerer P Theis FJ. SCANPY: large-scale single-cell gene expression data analysis. Genome Biol (2018) 19:15. doi: 10.1186/s13059-017-1382-0 29409532 PMC5802054

[69] Pedregosa F Varoquaux G Gramfort A Michel V Thirion B Grisel O. Scikit-learn: Machine learning in Python. Journal of Machine Learning Research (2012) 12, 2825–2830. doi: 10.48550/arXiv.1201.0490

[70] Korsunsky I Millard N Fan J Slowikowski K Zhang F Wei K. Fast, sensitive and accurate integration of single-cell data with harmony. Nat Methods (2019) 16:1289–96. doi: 10.1038/s41592-019-0619-0 PMC6884693 31740819

[71] Mereu E Lafzi A Moutinho C Ziegenhain C McCarthy DJ Álvarez-Varela A. Benchmarking single-cell RNA-sequencing protocols for cell atlas projects. Nat Biotechnol (2020) 38:747–55. doi: 10.1038/s41587-020-0469-4 32518403

[72] Kanehisa M. Toward understanding the origin and evolution of cellular organisms. Protein Sci (2019) 28:1947–51. doi: 10.1002/pro.3715 PMC6798127 31441146

[73] Schaefer CF Anthony K Krupa S Buchoff J Day M Hannay T. PID: the pathway interaction database. Nucleic Acids Res (2009) 37: Issue suppl_1 D674–D679. doi: 10.1093/nar/gkn653 PMC2686461 18832364

[74] Fabregat A Jupe S Matthews L Sidiropoulos K Gillespie M Garapati P. The reactome pathway knowledgebase. Nucleic Acids Res (2018) 46:D649–55. doi: 10.1093/nar/gkx1132 PMC5753187 29145629

[75] Liberzon A Birger C Thorvaldsdóttir H Ghandi M Mesirov JP Tamayo P. The molecular signatures database (MSigDB) hallmark gene set collection. Cell Syst (2015) 1:417–25. doi: 10.1016/j.cels.2015.12.004 PMC4707969 26771021

[76] Nishimura D. BioCarta. Biotech Softw Internet Rep (2001) 2:117–20. doi: 10.1089/152791601750294344

[77] The Gene Ontology Consortium. The gene ontology resource: 20 years and still GOing strong. Nucleic Acids Res (2019) 47:D330–8. doi: 10.1093/nar/gky1055 PMC6323945 30395331

[78] Ashburner M Ball CA Blake JA Botstein D Butler H Cherry JM. Gene ontology: Tool for the unification of biology. Nat Genet (2000) 25:25–9. doi: 10.1038/75556 PMC3037419 10802651

[79] Slenter DN Kutmon M Hanspers K Riutta A Windsor J Nunes N. WikiPathways: A multifaceted pathway database bridging metabolomics to other omics research. Nucleic Acids Res (2018) 46:D661–7. doi: 10.1093/nar/gkx1064 PMC5753270 29136241

[80] Wang C Wagner A Fessler J Avila-Pacheco J Karminski J Thakore P. Metabolic and epigenomic regulation of th17/treg balance by the polyamine pathway. BioRxiv (2020). doi: 10.1101/2020.01.23.911966

[81] Thiele I Swainston N Fleming RMT Hoppe A Sahoo S Aurich MK. A community-driven global reconstruction of human metabolism. Nat Biotechnol (2013) 31:419–25. doi: 10.1038/nbt.2488 PMC3856361 23455439

[82] DeTomaso D Jones MG Subramaniam M Ashuach T Ye CJ Yosef N. Functional interpretation of single cell similarity maps. Nat Commun (2019) 10:4376. doi: 10.1038/s41467-019-12235-0 31558714 PMC6763499

[83] Thiele I Palsson BØ. A protocol for generating a high-quality genome-scale metabolic reconstruction. Nat Protoc (2010) 5:93–121. doi: 10.1038/nprot.2009.203 20057383 PMC3125167

[84] Noronha A Modamio J Jarosz Y Guerard E Sompairac N Preciat G. The virtual metabolic human database: integrating human and gut microbiome metabolism with nutrition and disease. Nucleic Acids Res (2019) 47:D614–24. doi: 10.1093/nar/gky992 PMC6323901 30371894

[85] Scialdone A Natarajan KN Saraiva LR Proserpio V Teichmann SA Stegle O. Computational assignment of cell-cycle stage from single-cell transcriptome data. Methods (2015) 85:54–61. doi: 10.1016/j.ymeth.2015.06.021 26142758

[86] Lun ATL McCarthy DJ Marioni JC. A step-by-step workflow for low-level analysis of single-cell RNA-seq data with bioconductor. [version 2; peer review: 3 approved, 2 approved with reservations]. F1000Res (2016) 5:2122. doi: 10.12688/f1000research.9501.2 27909575 PMC5112579

[87] Hänzelmann S Castelo R Guinney J. GSVA: gene set variation analysis for microarray and RNA-seq data. BMC Bioinf (2013) 14:7. doi: 10.1186/1471-2105-14-7 PMC3618321 23323831

[88] Conway JR Lex A Gehlenborg N. UpSetR: an r package for the visualization of intersecting sets and their properties. Bioinformatics (2017) 33:2938–40. doi: 10.1093/bioinformatics/btx364 PMC5870712 28645171

[89] Aibar S González-Blas CB Moerman T Huynh-Thu VA Imrichova H Hulselmans G. SCENIC: single-cell regulatory network inference and clustering. Nat Methods (2017) 14:1083–6. doi: 10.1038/nmeth.4463 PMC5937676 28991892

[90] Durinck S Moreau Y Kasprzyk A Davis S De Moor B Brazma A. BioMart and bioconductor: A powerful link between biological databases and microarray data analysis. Bioinformatics (2005) 21:3439–40. doi: 10.1093/bioinformatics/bti525 16082012

[91] Kim K Shim D Lee JS Zaitsev K Williams JW Kim K-W. Transcriptome analysis reveals nonfoamy rather than foamy plaque macrophages are proinflammatory in atherosclerotic murine models. Circ Res (2018) 123:1127–42. doi: 10.1161/CIRCRESAHA.118.312804 PMC6945121 30359200

[92] Soneson C Robinson MD. Bias, robustness and scalability in single-cell differential expression analysis. Nat Methods (2018) 15:255–61. doi: 10.1038/nmeth.4612 29481549

[93] Mou T Deng W Gu F Pawitan Y Vu TN. Reproducibility of methods to detect differentially expressed genes from single-cell RNA sequencing. Front Genet (2019) 10:1331. doi: 10.3389/fgene.2019.01331 32010190 PMC6979262

[94] Robinson MD McCarthy DJ Smyth GK. edgeR: A bioconductor package for differential expression analysis of digital gene expression data. Bioinformatics (2010) 26:139–40. doi: 10.1093/bioinformatics/btp616 PMC2796818 19910308

[95] Reimand J Kull M Peterson H Hansen J Vilo J. g:Profiler–a web-based toolset for functional profiling of gene lists from large-scale experiments. Nucleic Acids Res (2007) 35:W193–200. doi: 10.1093/nar/gkm226 PMC1933153 17478515

[96] Yu G Wang L-G Han Y He Q-Y. clusterProfiler: an r package for comparing biological themes among gene clusters. OMICS (2012) 16:284–7. doi: 10.1089/omi.2011.0118 PMC3339379 22455463

[97] Vento-Tormo R Efremova M Botting RA Turco MY Vento-Tormo M Meyer KB. Single-cell reconstruction of the early maternal-fetal interface in humans. Nature (2018) 563:347–53. doi: 10.1038/s41586-018-0698-6 PMC7612850 30429548

[98] Bonnardel J T’Jonck W Gaublomme D Browaeys R Scott CL Martens L. Stellate cells, hepatocytes, and endothelial cells imprint the kupffer cell identity on monocytes colonizing the liver macrophage niche. Immunity (2019) 51:638–654.e9. doi: 10.1016/j.immuni.2019.08.017 31561945 PMC6876284

[99] Luecken MD Theis FJ. Current best practices in single-cell RNA-seq analysis: a tutorial. Mol Syst Biol (2019) 15:e8746. doi: 10.15252/msb.20188746 31217225 PMC6582955

[100] Muus C Luecken MD Eraslan G Waghray A Heimberg G Sikkema L. Integrated analyses of single-cell atlases reveal age, gender, and smoking status associations with cell type-specific expression of mediators of SARS-CoV-2 viral entry and highlights inflammatory programs in putative target cells. BioRxiv (2020). doi: 10.1101/2020.04.19.049254

[101] Wickham H. ggplot2 - elegant graphics for data analysis. 2nd ed. Cham: Springer International Publishing (2016). doi: 10.1007/978-3-319-24277-4

