# Supplementary figures and images for: Alveolar macrophages in early stage COPD show functional deviations with properties of impaired immune activation
Source: Front Immunol. 2022 Jul 28;13:917232. doi: 10.3389/fimmu.2022.917232 (PMC9377018; doi:10.3389/fimmu.2022.917232)

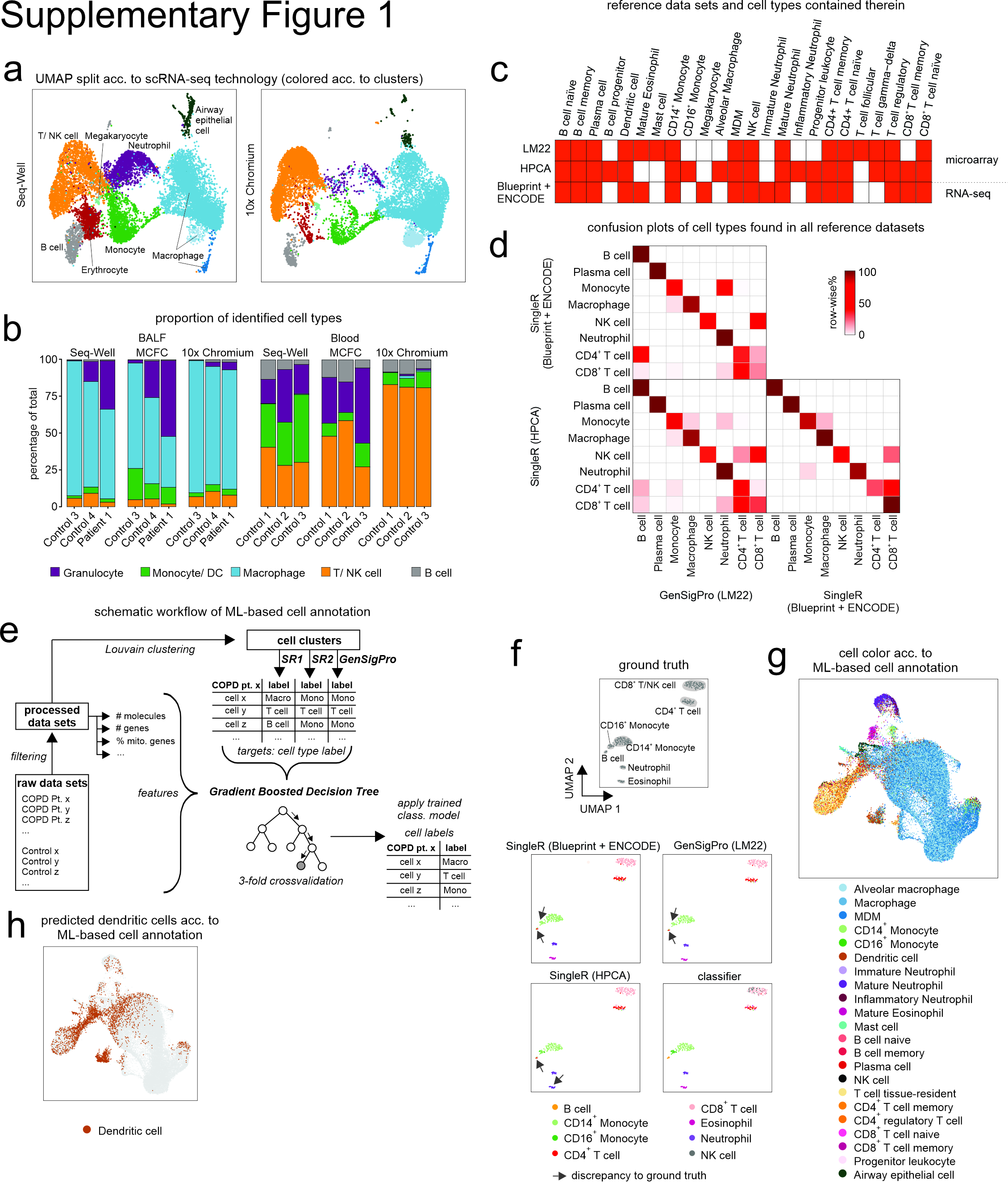

Supplement: Supplementary Figure 1 — Characterization of BALF immune cells using appropriate scRNA-seq technology and four-step cell-type annotation strategy (related to ). (A) UMAP representation of integrated blood and BALF data from different patients and two scRNA-seq technologies (10x Chromium and Seq-Well). The UMAP is split by technology and colored according to identified clusters. Clusters were additionally assigned to cell-types based on marker gene expression (according to Table S6). (B) Stacked bar plots of the relative cell-type proportions for MCFC, which served as ground truth, and cell-type proportions based on the assigned clusters of the two scRNA-seq technologies. (C) Overview of the cell types contained in the reference files used for cell-type annotation. The orange color indicates that the respective cell type is included in the reference file. (D) Confusion plots showing the concordance between the respective cell-type annotations across different annotation methods (SingleR with the Blueprint + ENCODE or HPCA as reference and GenSigPro with signatures from the LM22 dataset as reference). Only cell types that can be found in all reference files as shown in Figure S1C are displayed. (E) Scheme of the gradient boosted decision tree-based machine learning-approach for cell-type annotation. (F) UMAP representation of a benchmarking blood immune cell dataset (according to Table S1). The cells in the UMAPs are colored according to the respective cell annotation methods. The ground truth is derived based on the unique cell-type marker gene expression of each cell. Accumulation of cells that are annotated by the respective annotation methods, but show a deviation in the annotation with respect to the ground truth, are marked with an arrow. (G) UMAP representation of integrated BALF data obtained from COPD patients and control donors via the Seq-Well technology. Coloring according to machine learning-based cell-type annotation. (H) UMAP representation of the integrated dataset with the co [file Image_1.jpeg]

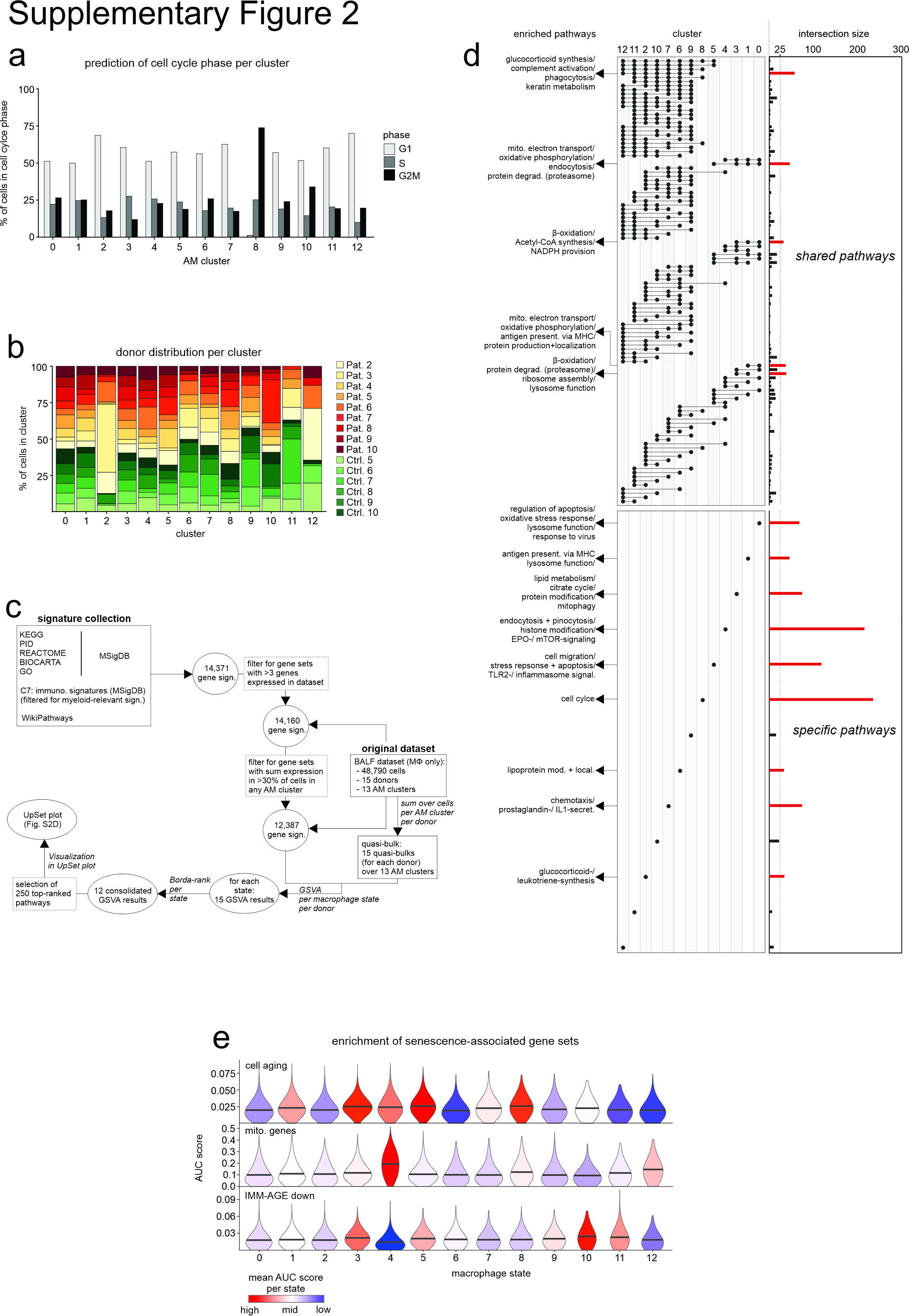

Supplement: Supplementary Figure 2 — Characterization of identified macrophage states (related to ). (A) Bar plot representation of the proportion of cells in the respective cell cycle states per cluster (according to ). (B) Stacked bar plot showing the proportion of individual donors in each macrophage state. (C) Schematic workflow to predict the cellular functions of each cluster based on gene set variation analysis (GSVA). (D) UpSet plot of the GSVA results (according to Figure S2C). Terms of cellular functions found in the same clusters are grouped into bins and the size of the bins is represented as a bar plot on the right, with bins containing more than 25 terms (dashed line) colored red. On the left side, dots indicate which clusters contain and share the binned terms. Frequently occurring terms of cellular functions within the bins containing more than 25 terms are shown. (E) Violin plots (with marked median enrichment values) displaying enrichment of different gene sets across clusters based on the Area Under the Curve (AUC). BALF = bronchoalveolar lavage fluid; sign. = signature; MФ = macrophage; GSVA = gene set variation analysis; mito. = mitochondrial; degrad. = degradation; mod. = modification; present. = presentation. [file Image_2.jpeg]

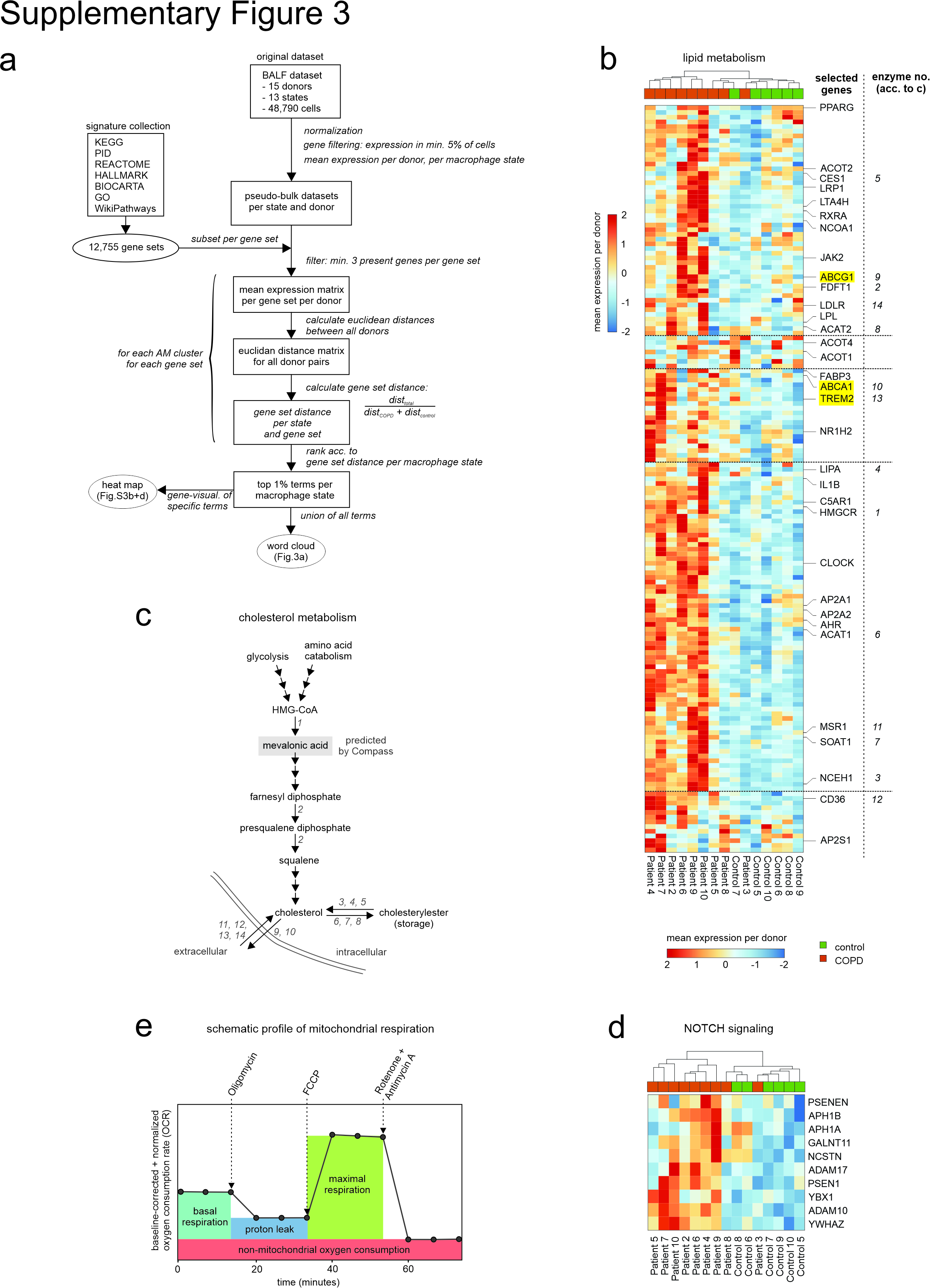

Supplement: Supplementary Figure 3 — Characterization of altered lipid metabolism in macrophages of COPD patients (related to ). (A) Schematic workflow of the GO-shuffling approach. (B) Heat map of lipid metabolism-associated genes predicted by the GO-shuffling approach. The mean gene expression per donor is represented as a z-transformed value (across all donors). Columns and rows of the heat map are sorted by hierarchical clustering. Genes that have been described as causing cholesteryl accumulation through the dysfunctionality of their protein products are marked in yellow. (C) Schema of the key steps in cholesterol metabolism and storage. Metabolites predicted by Compass are highlighted with a gray background. Enzymes involved in metabolism are abbreviated with a number that identifies them also in Figure S3B. (D) Heat map of NOTCH-signaling associated genes predicted by the GO-shuffling approach. The mean gene expression per donor is represented as a z-transformed value (across all donors). Columns and rows of the heat map are sorted by hierarchical clustering. (E) Schema of the time-dependent course of the oxygen consumption rate (OCR) and the inferred mitochondrial parameters based on the injection of different compounds (shown at the top of the plot). dist = distance; visual = visualization. [file Image_3.jpeg]

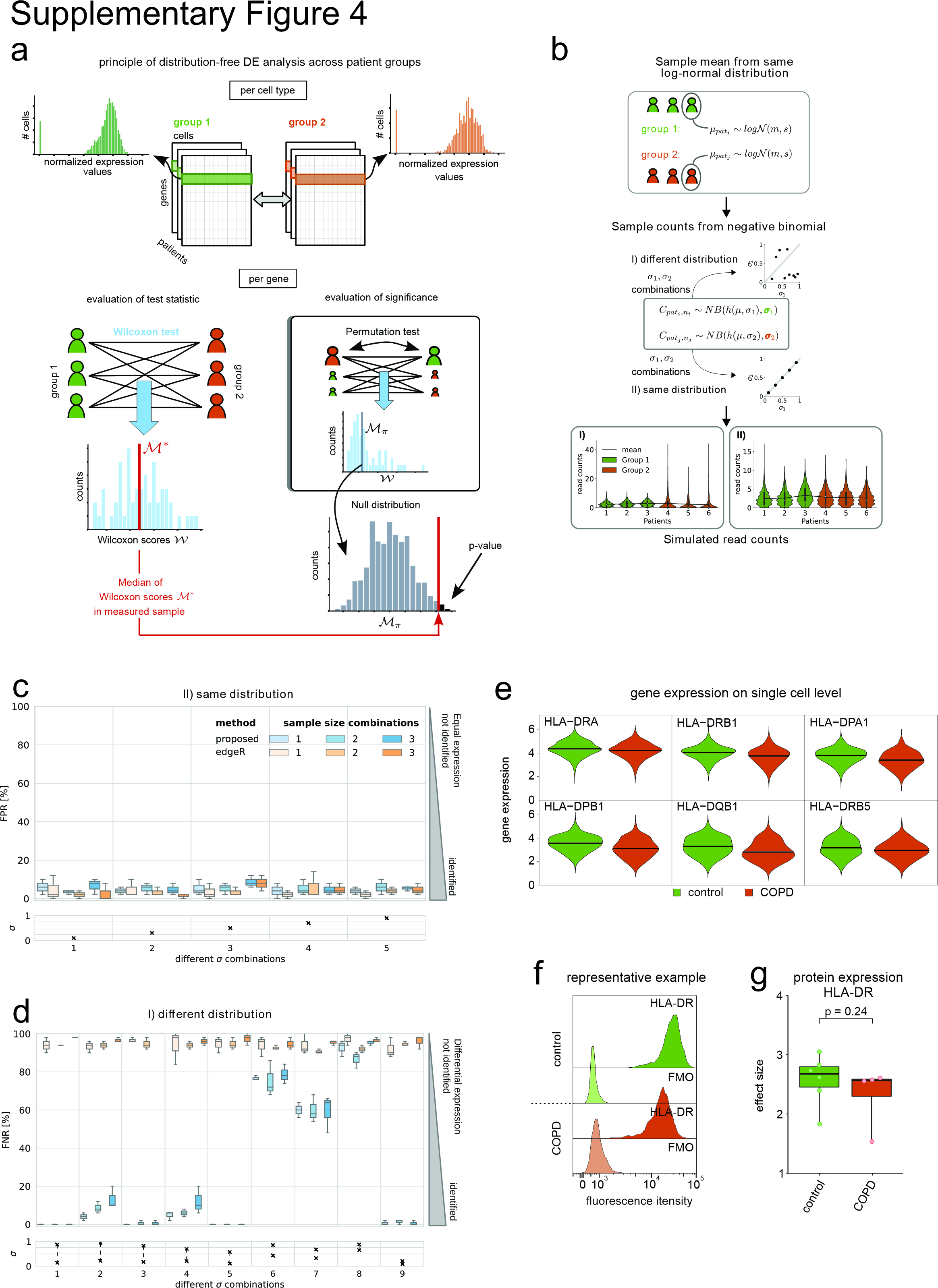

Supplement: Supplementary Figure 4 — Benchmarking of the novel DE-analysis approach (related to ). (A) Schematic workflow of the permutation test-based DE analysis approach. (B) Workflow for the simulation study used for the evaluation of the performance for the proposed DE-method. Two cases are considered, single cell count data was simulated for multiple patients within two groups, with I) different distributions between the groups, II) similar distributions between the groups. For each patient, the mean of the read counts is sampled from the same log-normal distribution with m = 1 and s = 0.15. The read counts are sampled from a negative binomial distribution with success probability (σ) parameters dependent on the considered case. When simulating different distributions between the groups (I), distinct σ-values are assigned to each group. When simulating the same distributions for the groups (II), the same σ-values are assigned to the groups. The number of cells per patient were chosen according to the Seq-Well dataset, where three sample size combinations were considered, originating from three macrophage state datasets. (C) False positive rate (FPR) in simulation study (II) (percentage of genes with a p-value < 0.05) for the proposed method and edgeR for 5 σ combinations and for each sample size realization. Boxplots (with marked median values) comprise results of three repeated sets of simulated data (one set of simulated data: 50* μ per patient, per σ combination). Low FPR denotes a higher number of correct equally expressed gene classifications. (D) False negative rate (FNR) in simulation study (I) (percentage of genes with a p-value > 0.05) for the proposed method and edgeR for 9 σ-combinations and for each sample size realization. Boxplots (with marked median values) comprise results of three repeated sets of simulated data (one set of simulated data: 50* μ per patient, per σ combination). Low FNR denotes a higher number of correct differential expressed gene classifications. (E) Violin plot [file Image_4.jpeg]

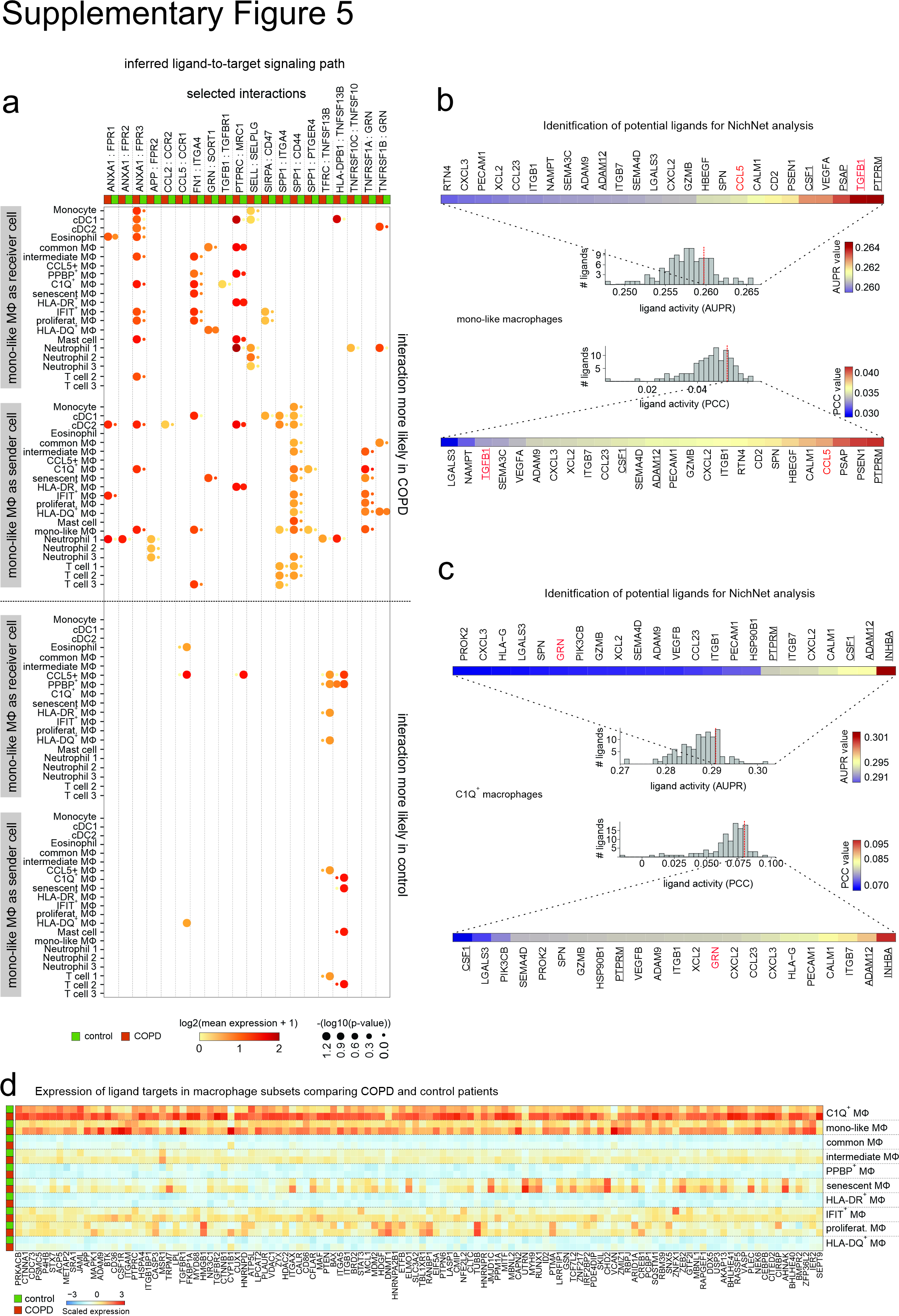

Supplement: Supplementary Figure 5 — Investigation of cell-to-cell interactions to infer important signaling pathways in macrophages (related to ). (A) Dot plot representation of monocyte-like macrophage-dependent ligand-receptor interactions predicted by CellPhoneDB that show significant enrichment (represented by the p-value) of the interacting pair in the interacting cell types either in COPD or in the control. Depicted are only selected interactions. (B, C) Illustration of the selection of potential upstream ligands of monocyte-like macrophages or C1Q+ macrophages based on the NicheNet analysis. The histograms show distributions based on ligand activity derived from the area under the precision recall curve (AUPR, upper histogram) and the Pearson correlation coefficient (PCC, lower histogram). The ligand activity of the highest ranked ligands is displayed in a color code together with the names of the 20 highest ranked ligands. The ligands predicted by the CellPhoneDB analysis (according to Figure S5A) are highlighted in red and the top 3 ligands based on AUPR for either monocyte-like macrophages or C1Q+ macrophages (as presented in ) are underlined. (D) Expression of ligand targets from in macrophage subsets comparing COPD and control patients (z-transformed by gene). AUPR = area under the precision recall curve; PCC = Pearson correlation coefficient; MФ = macrophage; DC = dendritic cell. [file Image_5.jpeg]

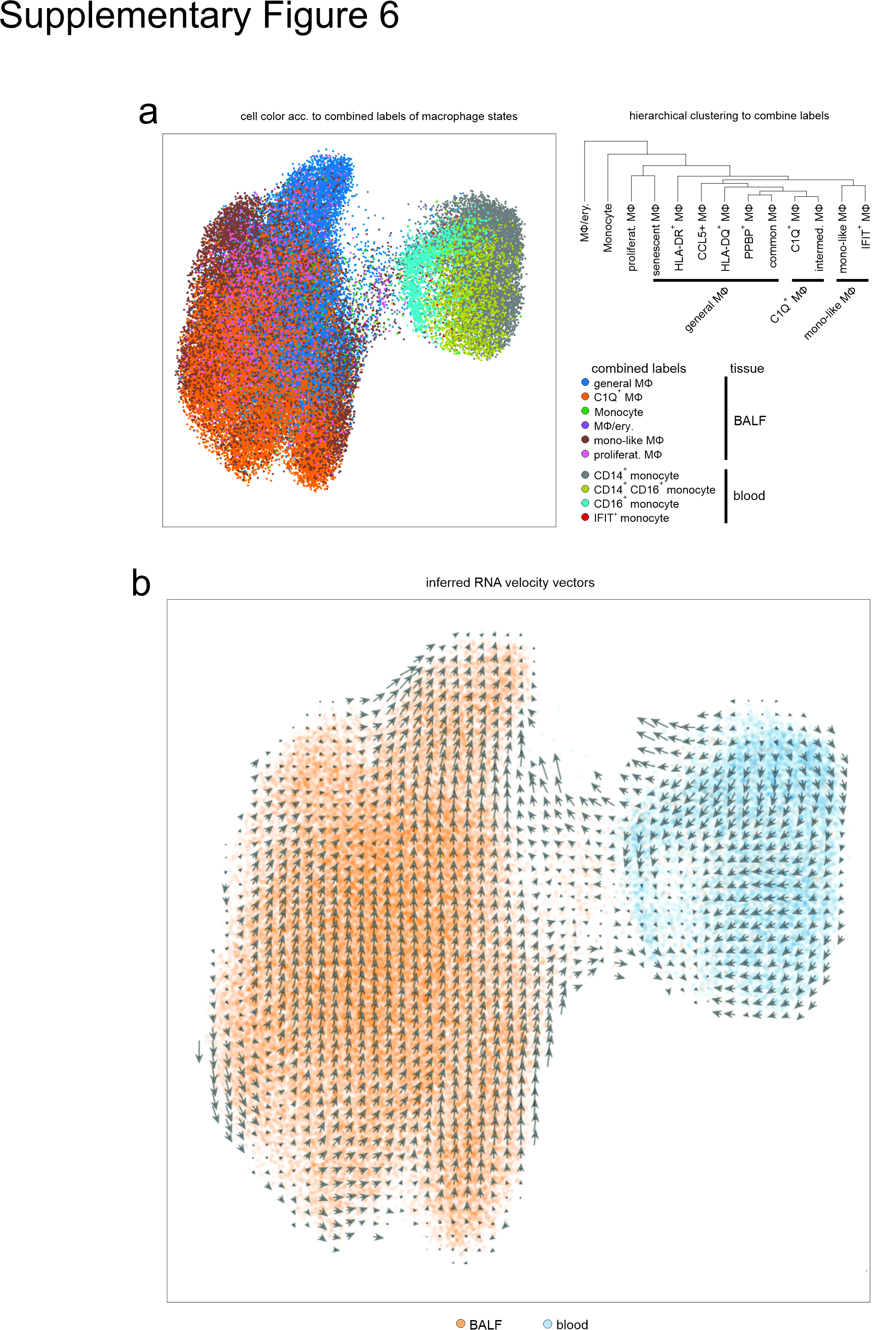

Supplement: Supplementary Figure 6 — Modeling the association of blood monocytes and BALF macrophages (related to ). (A) UMAP of embedded macrophages/monocytes from BALF and blood monocytes with coloring according to the cell types derived from the combined labels. The dendrogram on the right side illustrates the transcriptional relationship between the macrophage subtypes and shows how several subtypes were summarized in the combined labels. (B) Projection of computed RNA velocity vectors onto the UMAP of the embedded data. BALF = bronchoalveolar lavage fluid; mono = monocyte; MФ = macrophage; proliferat. = proliferating. [file Image_6.jpeg]

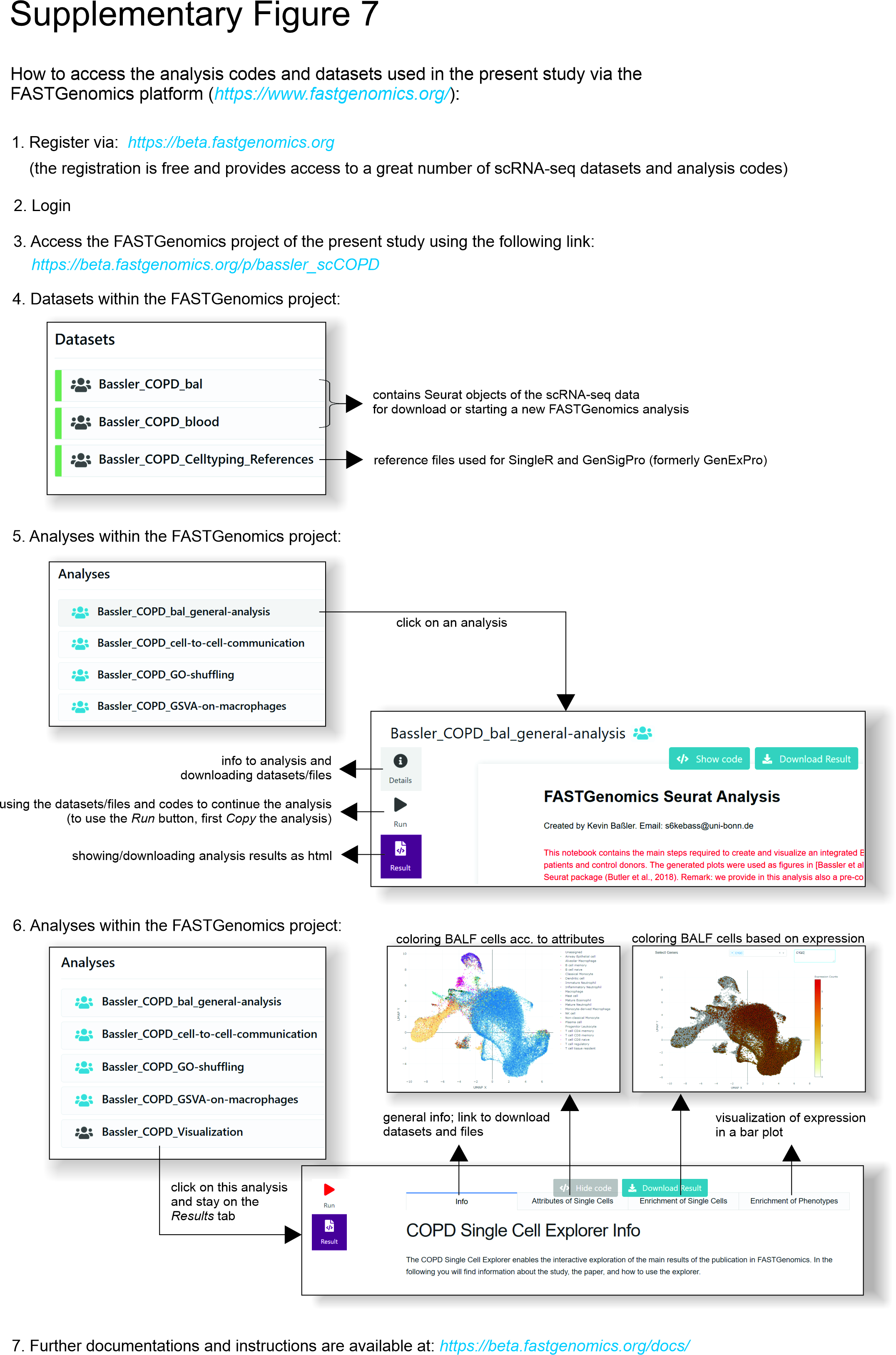

Supplement: Supplementary Figure 7 — Instructions for accessing the COPD Seq-Well dataset and scripts via the FASTGenomics platform. [file Image_7.jpeg]
